# Spontaneous mutation rate is a plastic trait associated with population density across domains of life

**DOI:** 10.1371/journal.pbio.2002731

**Published:** 2017-08-24

**Authors:** Rok Krašovec, Huw Richards, Danna R. Gifford, Charlie Hatcher, Katy J. Faulkner, Roman V. Belavkin, Alastair Channon, Elizabeth Aston, Andrew J. McBain, Christopher G. Knight

**Affiliations:** 1 Faculty of Science and Engineering, The University of Manchester, Manchester, United Kingdom; 2 Faculty of Biology, Medicine and Health, The University of Manchester, Manchester, United Kingdom; 3 School of Engineering and Information Sciences, Middlesex University, London, United Kingdom; 4 School of Computing and Mathematics, Keele University, Keele, United Kingdom; Massachusetts Institute of Technology, United States of America

## Abstract

Rates of random, spontaneous mutation can vary plastically, dependent upon the environment. Such plasticity affects evolutionary trajectories and may be adaptive. We recently identified an inverse plastic association between mutation rate and population density at 1 locus in 1 species of bacterium. It is unknown how widespread this association is, whether it varies among organisms, and what molecular mechanisms of mutagenesis or repair are required for this mutation-rate plasticity. Here, we address all 3 questions. We identify a strong negative association between mutation rate and population density across 70 years of published literature, comprising hundreds of mutation rates estimated using phenotypic markers of mutation (fluctuation tests) from all domains of life and viruses. We test this relationship experimentally, determining that there is indeed density-associated mutation-rate plasticity (DAMP) at multiple loci in both eukaryotes and bacteria, with up to 23-fold lower mutation rates at higher population densities. We find that the degree of plasticity varies, even among closely related organisms. Nonetheless, in each domain tested, DAMP requires proteins scavenging the mutagenic oxidised nucleotide 8-oxo-dGTP. This implies that phenotypic markers give a more precise view of mutation rate than previously believed: having accounted for other known factors affecting mutation rate, controlling for population density can reduce variation in mutation-rate estimates by 93%. Widespread DAMP, which we manipulate genetically in disparate organisms, also provides a novel trait to use in the fight against the evolution of antimicrobial resistance. Such a prevalent environmental association and conserved mechanism suggest that mutation has varied plastically with population density since the early origins of life.

## Author summary

Spontaneous mutations fuel evolution, but the rate at which they occur can vary for a particular organism depending on its environment—a phenomenon known as mutation-rate plasticity. For microbes growing in liquid, the density to which a population can grow is a key feature of the environment. We find that organisms’ mutation rates are associated with the density to which they grow, such that higher population densities are associated with lower mutation rates. Initially we identify this density-associated mutation-rate plasticity (DAMP) in data culled from the published literature: beyond well-known patterns of mutation-rate variation among organisms, we see substantial variation within diverse organisms, the large majority of which is associated with population densities. We test this association in the laboratory, finding DAMP at different sites in the genomes of both bacteria (*Escherichia coli*) and eukaryotes (the yeast, *Saccharomyces cerevisiae*). In each case, DAMP requires a protein that avoids mutation by cleaning cells of oxidatively damaged guanine nucleotides (MutT in *E*. *coli* and Pcd1 in yeast). In our assays, DAMP results in a lower probability of seeing the evolution of antibiotic resistance at higher population densities. We anticipate that DAMP affects the course of evolution more generally and that understanding its causes and effects will help us to understand and control evolutionary trajectories.

## Introduction

The probability of spontaneous genetic mutations occurring during replication evolves among organisms [1]. This mutation rate can also vary at a particular site in a particular genotype, dependent upon the environment [2]. Specifically, mutation rate can increase with endogenous and exogenous factors [3]. Indeed, any factor that affects the balance between mutagenesis and DNA repair can modify the mutation rate. These include intracellular nucleotide pools [4], organism age [5], and factors affecting the expression [6] and stochastic presence or absence [7] of low copy number repair proteins. Where such mutation/repair-balance factors depend on the environment, the result is mutation-rate plasticity. Plastic mutation rates have been most thoroughly addressed for stress-induced mutagenesis. This may involve the induction of error-prone polymerases, for instance, in the *E*. *coli* SOS response [8]. We have recently identified a novel mode of mutation-rate plasticity in response to population density in *E*. *coli*. This plasticity does not have any very obvious association with stress—the densest populations, experiencing the most competition, show the lowest mutation rates [9].

Understanding mutation-rate plasticity is hampered by the difficulty of accurately measuring any mutation rate. Spontaneous mutation rates have long been estimated in microbes using counts of cells gaining a phenotypic marker of mutation in environments lacking selection for that marker: the “fluctuation test” created by Luria and Delbrück in 1943 [10]. Alternative approaches to measuring rates of mutation in the absence of selection, such as accumulation of mutations through many population bottlenecks [11], directly comparing genome sequences of parents and offspring [12], or targeted population sequencing [13], are much more laborious and thus poorly suited to potentially dynamic responses. Therefore, well-conducted fluctuation tests [14], remain the most appropriate tool to assay environmental dependence in mutation rates.

Population density affects many traits, particularly in microbes [15]. Its association with mutation rate has great potential to affect evolutionary trajectories [16] in ways relevant to the evolution of antimicrobial resistance [9]. However, thus far, this plasticity associated with population density is poorly understood: Its prevalence across domains of life is unknown. Whether it varies among organisms, enabling its evolution, remains to be tested. A little is known about the relationship of mutation rate with density perception in 1 organism [17], but the required downstream mechanisms of mutation or repair remain uncharacterised. Here, we address each of these issues. We demonstrate that there is indeed density-associated mutation-rate plasticity (DAMP) across domains of life: high population density is associated with low mutation rates. DAMP differs between closely related organisms, indicating that this trait does indeed evolve. Strikingly, the same mutation avoidance mechanism is required to modulate mutation rate in response to population density in both prokaryotes and eukaryotes.

## Results

**Fig 1:**
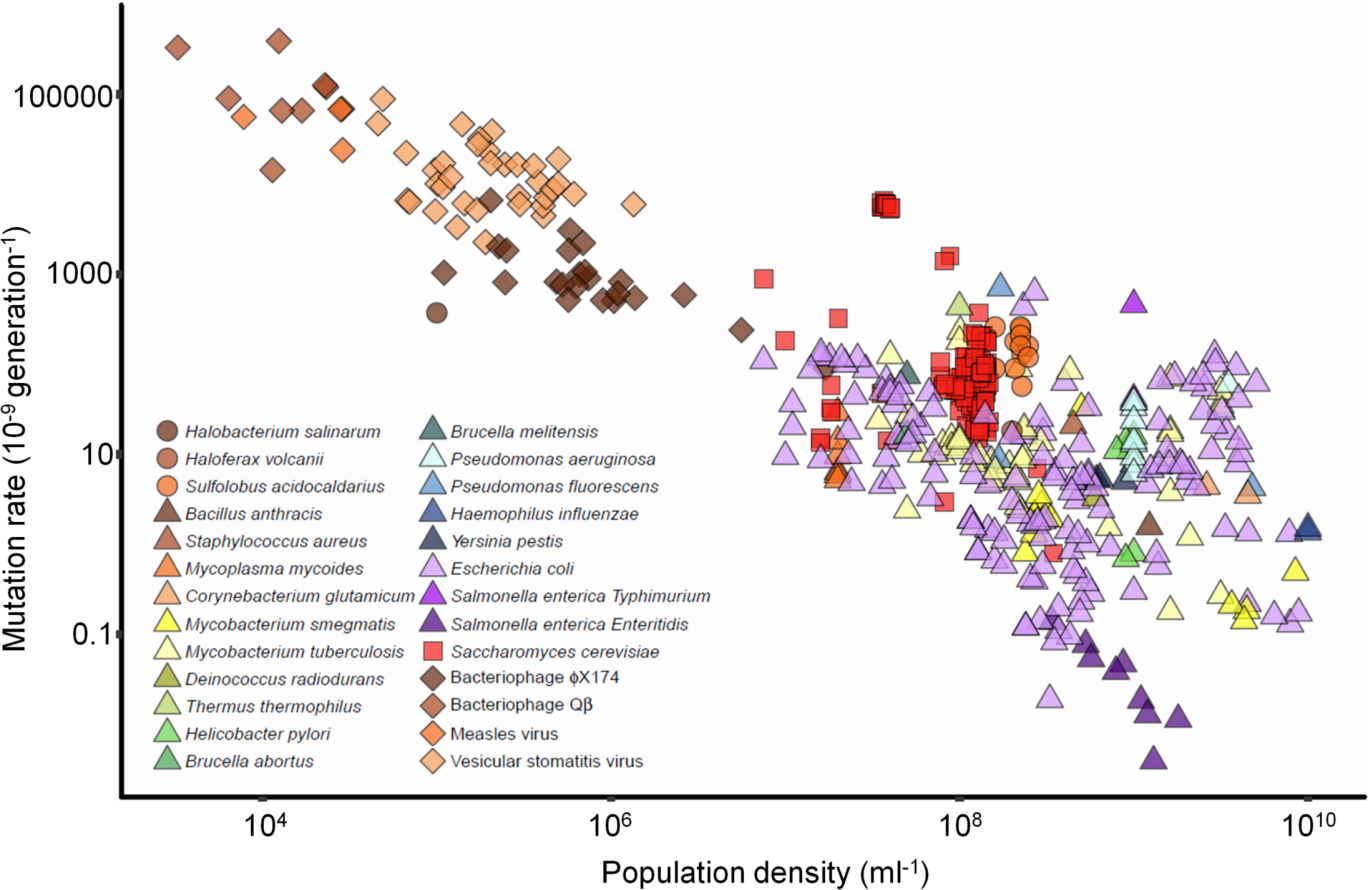
Mutation rates published from 1943 to 2017 in relation to population density. Colour indicates organism, and shape indicates domain: Archaea (circle), Bacteria (triangle), Eukaryota (square), and viruses/bacteriophage (diamond). Mutation rates were estimated using a wide variety of phenotypic markers (*N* = 70). The genetic basis and number of mutations giving each phenotype varies and is not known for all markers (i.e., different mutation rates may be generated from the same underlying per base-pair rate). Population densities are estimated by different techniques (cell counts, colony-, or plaque-forming units). See Results, Materials and Methods, and Model S-I in S1 Text for statistical analysis accounting for these and other differences. Note the logarithmic axes. Raw data is available in S1 Data.

In order to test the nature and prevalence of mutation-rate plasticity, we considered over 70 years of published mutation rates estimated by fluctuation test. We collated 474 individual mutation-rate estimates from 68 independent studies that conducted fluctuation tests in organisms of 26 species from all domains of life and viruses, in which the final population density (*D*) an organism’s population reached was either reported or could be obtained from the original authors (Fig 1 and S1 Table).

There is a clear negative association between mutation rate and *D* (Spearman’s *ρ* = −0.66), spanning more than 6 orders of magnitude in both measures. This association potentially involves both between- and within-organism variation in mutation rate. We therefore analysed this relationship using a linear mixed-effects model (Model S-I in S1 Text), accounting for various features of the original experiments (organism, culture media, phenotypic marker, publication, and phylogenetic relationships among organisms). We find that, having taken all these factors into account, changes in *D* explain 93% of variation in published mutation-rate estimates (*N =* 474, *LR*_1_ = 22, *P =* 2.9×10^−6^; Model S-I in S1 Text). The model identifies substantial variation between-organisms in a negative within-organism association of mutation rate with *D* (slope varies from −0.46 to −0.98; S2 Fig). The average slope across organisms is −0.67 (−0.89 to −0.48 CI), meaning that mutation rate doubles with a 64% reduction in *D* (54% to 76% CI), quantitatively similar to the plasticity reported for *E*. *coli* B strains [9], in which the figure is 77% (61% to 96% CI).

Despite the striking association shown in Fig 1, the relationship could originate in various processes, including, but not limited to, mutation-rate plasticity. We consider several hypotheses: 1. **Technical bias**: (1a) The same estimate of final population size (*N*_*t*_) is typically used to calculate both *D* and the mutation rate. Therefore, any error in *N*_*t*_ could itself lead to a negative association between mutation rate and *D*. (1b) Fluctuation tests typically assume that the phenotypic markers used are selectively neutral; however, in practice, this is not always the case. If cultures grown to higher *D* also, typically, go through more generations, a systematic tendency towards phenotypic markers being costly could lead to underestimated mutation rates, specifically at high *D*. 2. **Reporting bias**: there could be an underrepresentation of reported low mutation rates at low *D* and high mutation rates at high *D*. This is expected because standard volume microbial cultures with low *D* may not have sufficient mutational events at marker loci to achieve good estimates of low mutation rates. Similarly, in dense populations, high mutation rates can produce more mutants than it is practical to count. 3. **DAMP**: the relationship in Fig 1 is consistent with DAMP across domains of life. However, the data in Fig 1 comes from diverse studies using very different experimental and analytical set-ups. It remains for us to test the association at different marker loci in different organisms within a single experimental and analytical framework.

**Fig 2:**
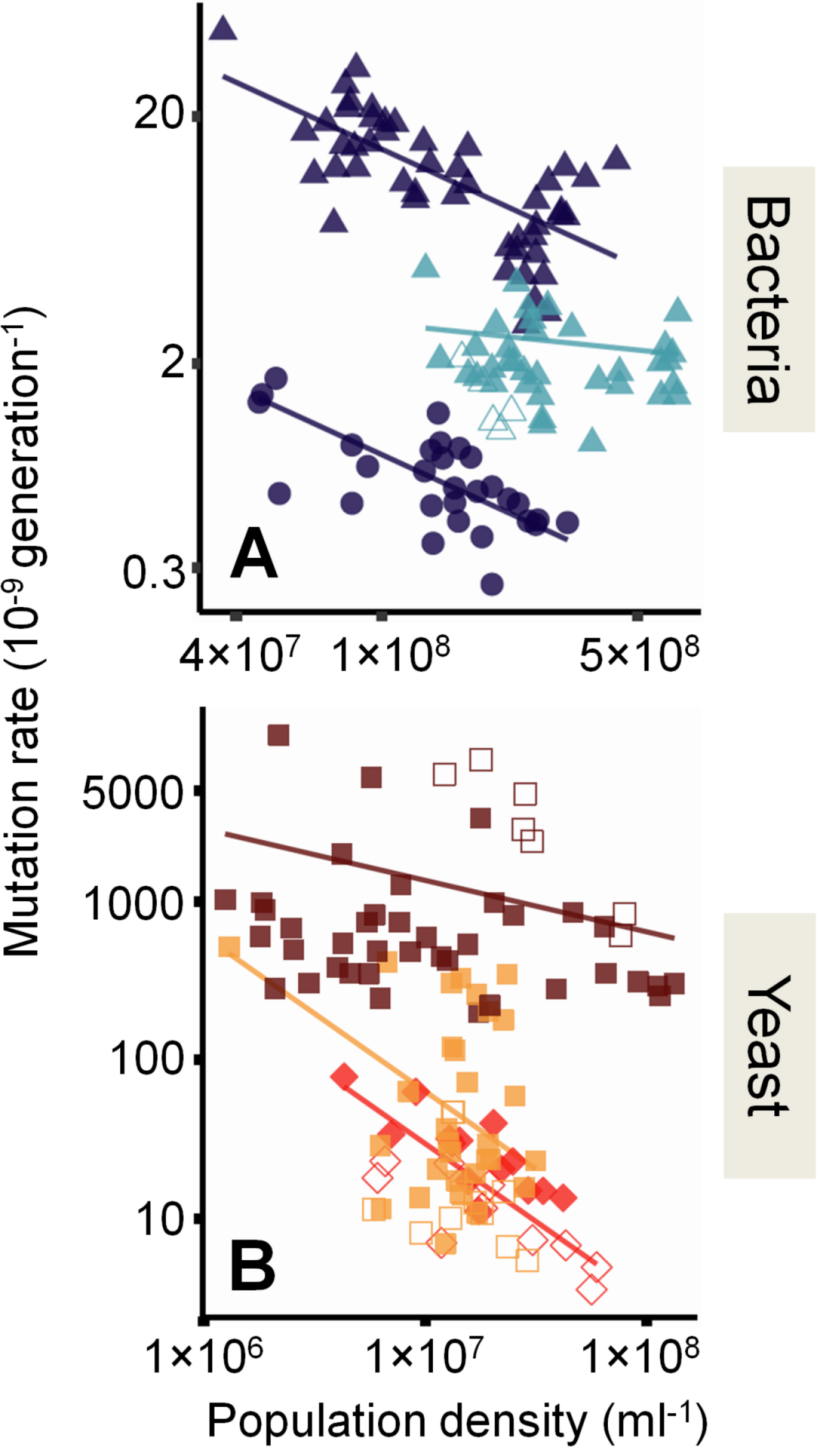
Density-associated mutation-rate plasticity (DAMP) in bacteria and yeast. (**A**) Mutation rates to rifampicin (triangles) and nalidixic acid (circles) resistance in *E*. *coli* MG1655 (dark blue, *N =* 77) and *P*. *aeruginosa* PAO1 (light blue, *N =* 40); lines from Model S-II in S1 Text: Wald test *t*_80_ = 11; *P =* 1.7×10^−18^ that *E*. *coli* slope is 0; *t*_80_ = 1.2, *P =* 0.22 that *P*. *aeruginosa* slope is 0. Lines are plotted separately for the 2 *E*. *coli* markers because there are more mutations conferring rifampicin resistance (in the *rpoB* gene) than nalidixic acid resistance (in the *gyrA* gene), resulting in different overall rates. (**B**) Mutation rates to hygromycin B (squares) and 5-Fluoro-orotic acid (5-FOA, diamonds) resistance in *S*. *cerevisiae* BY4742 (brown, *N =* 46), Sigma1278b (orange, *N =* 41), and S288C (red, *N =* 24); lines from Model S-III in S1 Text: *t*_81_ = 9.6; *P =* 4.3×10^−15^ Wald test that average *S*. *cerevisiae* slope with *D* is 0; likelihood ratio test that slopes are equal among strains *LR*_1_ = 17, *P =* 4.1×10^−5^. Open shapes denote mutation-rate estimates that would typically be excluded because the estimated number of mutational events per culture, *m*, is either below 0.3 or above 30. Population densities estimated by ATP-based assay in (**A**) and direct cell counts in (**B**). See main text for the slope estimates of the given lines. Note the logarithmic axes. Raw data are available in S1 Data.

To test these hypotheses, we focused on the 2 most-diverged genetic model organisms in Fig 1: the bacterium *E*. *coli* (strain MG1655) and the eukaryotic yeast *S*. *cerevisiae* (strains S288C, BY4742 and Sigma1278b). Using fluctuation tests, we estimated mutation rates in batch cultures at 2 marker loci in each organism: *rpoB* and *gyrA* in *E*. *coli* and 25S ribosomal proteins and *URA3* in *S*. *cerevisiae* (see Materials and Methods). These confer resistance to antibiotics rifampicin, nalidixic acid, hygromycin B, and 5-Fluoro-orotic acid (5-FOA), respectively. In each case, we varied culture volume and added nutrients to give different *D* (see Materials and Methods). To test hypothesis 1a (correlation of errors), we estimated *N*_*t*_ by 2 independent methods for each organism: an ATP-based luminescence assay and colony-forming units (CFU) for bacteria, haemocytometer cell counts (CC) and CFU for yeast. Using CFU to estimate mutation rate (typical in Fig 1) and any of the 3 methods to estimate *D* (Fig 2 and S4 Fig), we find significant variation of mutation rates with changing *D*: mutation rate varies from 5-fold to 23-fold across both loci in all organisms, where mutation rate is lower at high *D* (Fig 2). This refutes hypothesis 1a that the broad association between population density and mutation rate (Fig 1) is caused by a correlation of errors.

To test hypothesis 1b (costs of marker mutations) for the association between population density and mutation rate, we first considered the fitness effects of resistance mutations. Others have found small negative effects (12% on average in *Pseudomonas aeruginosa* [18]) and sometimes positive effects on fitness of mutations at the *rpoB* locus considered in Fig 2A [19,20]. Consistent with this, the fitness effects of mutations in our experiments are, on average, close to neutral (S5 Fig) and, where present, are similar across population densities (S6 Fig). Secondly, we reanalysed the data in Fig 2 assuming different average fitness effects of resistance mutations. We find that, even assuming large fitness costs (>50%), there is only a small flattening of the negative slope (S5 Fig). Thus, it is highly unlikely that the differential growth of resistant strains is responsible for the slopes seen in Fig 2, refuting hypothesis 1b that the association in Fig 1 may be accounted for by technical bias driven by differential growth of mutant strains.

Typically, mutation rates at which the estimated number of mutational events per culture (*m*) is either too low (<0.3) or too high (>30) are excluded and may not be reported [14]. To test hypothesis 2 (reporting bias) we kept a close account of all estimates. Those 34 estimates that would typically be excluded are shown as open symbols in Fig 2. As expected, these points fall in either the lower left or upper right of the data. Nonetheless, the association between *D* and mutation rate is similar and highly significant with or without these data (−0.68 [−0.80 to −0.56 CI] versus −0.68 [−0.79 to −0.56 CI] in *E*. *coli* and, on average, −0.65 [−0.79 to −0.52 CI] versus −0.48 [−0.63 to −0.33] in *S*. *cerevisiae*), refuting hypothesis 2—that the negative association between population density and mutation rate in Fig 1 is caused by reporting bias. In contrast, our findings from different loci and different organisms within a single experimental and analytical framework are consistent with the pattern across the literature (Fig 1 and S7 Fig), strongly supporting hypothesis 3—that there is negative DAMP across the domains of life.

Our assays in *S*. *cerevisiae* identify significant variation in DAMP slope among strains (Fig 2B): the slope for S288C and Sigma1278b is −0.98 (−1.2 to −0.73 CI), whereas in BY4742, it is −0.32 (−0.40 to −0.25 CI). This limited difference in plasticity suggests that this trait may evolve. To investigate the extent of interorganism variation in DAMP, we tested another model organism, much more closely related to *E*. *coli*: *P*. *aeruginosa* PAO1 (both are gram-negative gamma Proteobacteria). We find *P*. *aeruginosa* has a greatly reduced DAMP slope relative to *E*. *coli*, not significantly different from 0 (Fig 2A and S4A Fig, slope estimate −0.15 [−0.40 to +0.095 CI], Model S-II in S1 Text). This indicates that, while DAMP is very widespread, it has evolved among closely related organisms.

**Fig 3:**
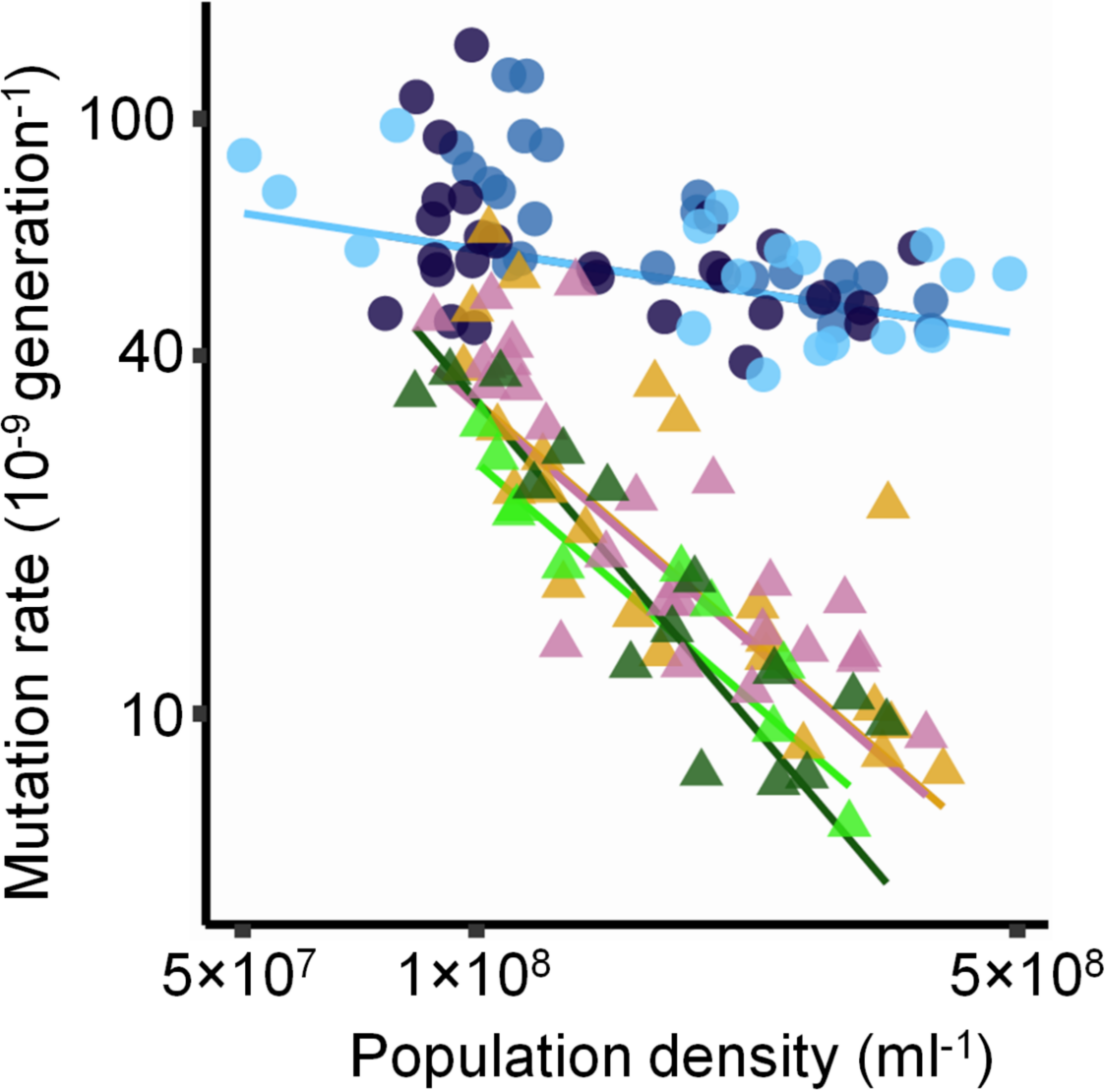
Density-associated mutation-rate plasticity (DAMP) in strains of *E*. *coli* with deficiencies in various DNA repair and other systems. Mutation rate in strains from the Keio collection [24] deleted for genes encoding deoxyadenosine methylase (Δ*dam*, *N =* 23, yellow triangles), error-prone DNA polymerase Pol IV (Δ*dinB*, *N =* 25, pink triangles), methionine transporter MetI (Δ*metI*, *N =* 10, light green triangles), endonuclease VIII (Δ*nei*, *N =* 15, dark green triangles), and each protein of methyl-directed DNA mismatch repair (MMR), which comprises MutS (Δ*mutS*, *N =* 28, light blue circles) that binds mismatched DNA and MutL (Δ*mutL*, *N =* 27, dark blue circles) that interacts with MutS to activate endonuclease MutH (Δ*mutH*, *N =* 24, medium blue circles). All lines result from Model S-VII in S1 Text. Wald tests that average slope for Δ*dam*, Δ*dinB*, and Δ*metI* is 0: *t*_92_ = 17; *P =* 4.2×10^−30^, that average slope for Δ*nei* is 0: *t*_91_ = 13; *P =* 6.0×10^−22^, and that average slope for MMR strains is 0: *t*_91_ = 4.3; *P =* 5.1×10^−5^. See S2 Table for further strain details. Densities estimated by ATP-based luminescence assay. As in Fig 2, triangles and circles indicate mutation rates to rifampicin and nalidixic acid resistance, respectively. Note the logarithmic axes. Raw data are available in S1 Data.

Diverse mechanisms, some broadly conserved, could in principle be modulated to give DAMP. These include polymerases used for DNA replication and repair, some of which are more error-prone than others [21], and systems that repair mutational mismatches [22] or remove mutagenic nucleotides before they can be incorporated into DNA [23]. To identify mechanisms by which the observed DAMP occurs, we tested *E*. *coli* strains deleted for genes involved in these processes (S2 Table). A strain lacking the error-prone polymerase Pol IV (encoded by *dinB*), implicated in stress-induced mutagenesis [8], displays DAMP (Fig 3; −1.1 [−1.3 to −1.00 CI]). *E*. *coli*’s methyl-directed DNA mismatch repair (MMR) system was hypothesised to be involved in DAMP [9]. Despite >100-fold increases in mutation rates, strains lacking MMR proteins (MutH, MutL, and MutS) still display DAMP, albeit with a less steep slope (Fig 3; −0.20 [−0.30 to −0.11 CI]). We also considered other systems potentially associated with DAMP: a strain lacking MetI (Fig 3), a transporter protein responsible for feeding methionine to the activated methyl cycle, which was previously implicated in DAMP [9]. Despite growing over only a relatively narrow range of densities, the *metI* deletant shows DAMP with a slope indistinguishable from the *dinB* deletant, as does a strain lacking Dam methylase, required for MMR to identify the ‘correct’ DNA strand (Fig 3). Finally, a strain lacking Endonuclease VIII (encoded by *nei* in *E*. *coli*) also shows DAMP, with the greatest slope among all these strains (Fig 3, −1.5 [−1.8 to −1.3 CI]).

**Fig 4:**
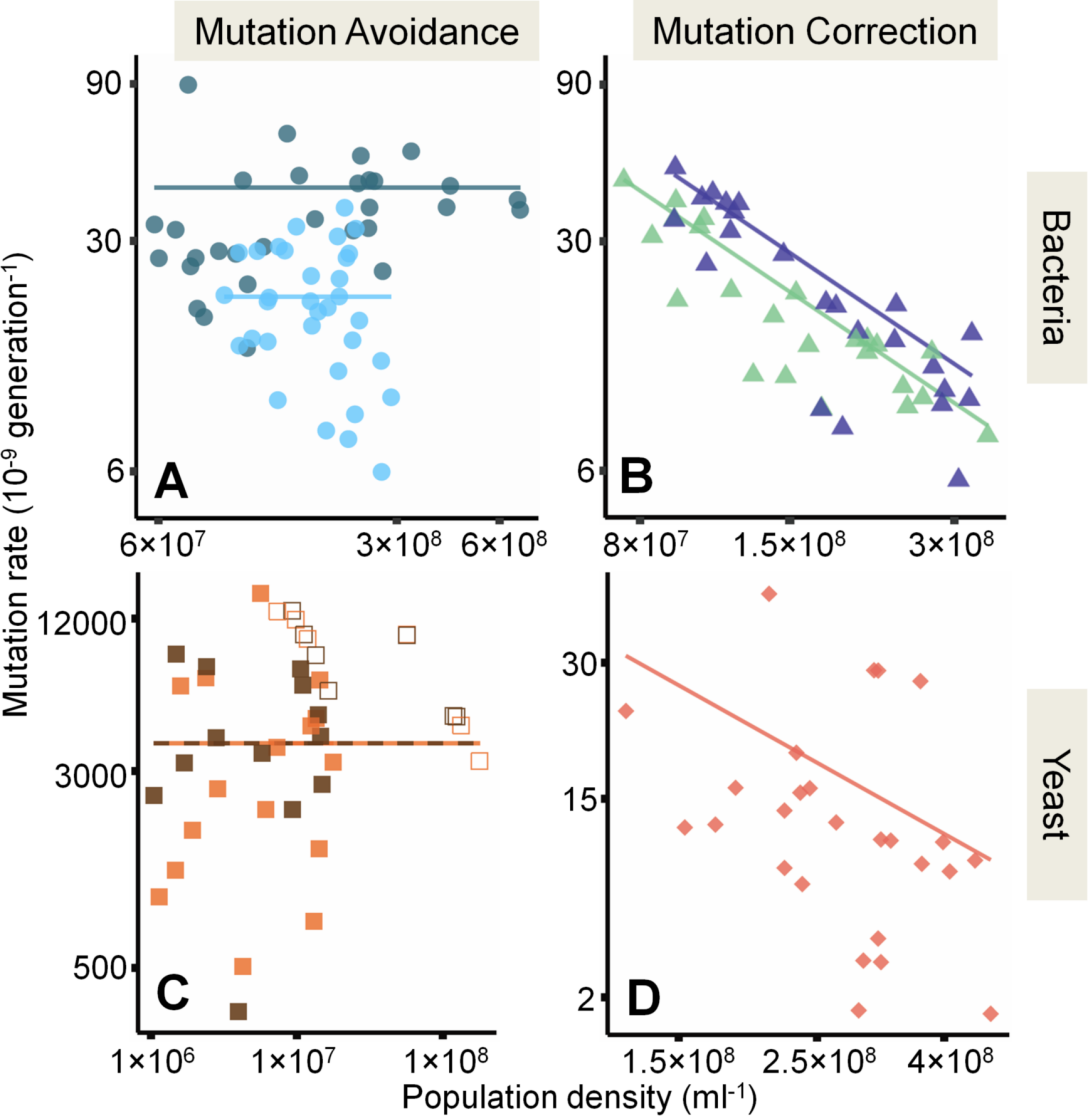
Density-associated mutation-rate plasticity (DAMP) in cells lacking mutation-avoidance or -correction genes in *E*. *coli* and *S*. *cerevisiae*. (**A**) Mutation rates to nalidixic acid resistance in 2 independent *E*. *coli* Δ*mutT* strains that share no secondary mutations (JW0097-1 and JW0097-3 S3 Table), dark and light blue, respectively (Model S-VIII in S1 Text) (**B**) Mutation rates to rifampicin resistance in *E*. *coli* Δ*mutM* and Δ*mutY* strains (medium blue and light green, respectively), with equal slopes −1.1 (−1.2 to −1.04, CI) (*N =* 46, Model S-X in S1 Text) (**C**) Mutation rate to hygromycin B resistance in *S*. *cerevisiae* BY4742 and Sigma1278b *PCD1-*Δ strains (brown and orange, respectively, Model S-XII in S1 Text) (**D**) Mutation rate to 5-Fluoro-orotic acid (5-FOA) resistance in *S*. *cerevisiae* Sigma1278b *MLH1-*Δ (slope −1.22 [−1.44 to −1.01 CI], *N =* 26, Model S-XIV in S1 Text). Open shapes are as in Fig 2. Population density measured by ATP-based luminescence assay in (**A**) and (**B**) and via colony-forming unit (CFU) counts in (**C**) and (**D**). See also the main text for the slope estimates of the given lines. Note the logarithmic axes. Raw data are available in S1 Data.

In contrast to the systems tested above, *mutT* deletion removed the dependence of mutation rate on *D* (Fig 4A and S8A Fig, likelihood ratio test of slope *N =* 63, *LR*_1_ = 1.5; *P =* 0.22, Model S-VIII in S1 Text). MutT is the mutation-avoidance component of the 8-Hydroxyguanine (GO) system, protecting cells from mutagenic effects of damaged nucleotides [23]. Specifically, the MutT Nudix hydrolase removes 8-oxo-dGTP from the free-nucleotide pool. This prevents AT to GC transversions, which occur when 8-oxo-dGTP is incorporated in place of a thymidine triphosphate nucleotide during DNA synthesis [25]. The GO system also includes mutation-correction proteins MutM and MutY, which target 8-oxo-dGTP once incorporated in DNA [23]. Strains lacking either protein display DAMP (Fig 4B and S8B Fig; Model S-X in S1 Text). Thus, while neither error-prone polymerase Pol IV nor the MMR system with Dam methylase is necessary (Fig 3), DAMP in *E*. *coli* (Fig 2A) requires scavenging the oxidised nucleotide 8-oxo-dGTP from the cellular pool.

Eukaryotes possess more, and more diverse, genes and systems for mutation avoidance and correction than bacteria. Nonetheless, the mutation-avoidance mechanism required for DAMP in *E*. *coli* is conserved across domains of life [26]. *PCD1* encodes the yeast 8-oxo-dGTPase functionally homologous to bacterial MutT [27]. We tested *PCD1-*Δ strains in 2 different *S*. *cerevisiae* backgrounds (BY4742 and Sigma1278b). As in *E*. *coli* Δ*mutT*, mutation rates are greatly increased in *PCD1-*Δ, with no evidence of DAMP (Fig 4C and S8C Fig likelihood ratio test of slope *N =* 42, *LR*_1_ = 0.18, *P =* 0.67, Model S-XII in S1 Text). In contrast, *MLH1*, a yeast gene homologous to *E*. *coli* MMR gene *mutL*, displays DAMP (Fig 4D and S8D Fig; Model S-XIV in S1 Text). Therefore, DAMP not only occurs across domains of life, but, in the bacteria and eukaryotes tested, requires the same specific mutation-avoidance mechanism.

## Discussion

The negative association between published mutation rates and population density (*D*, Fig 1) is remarkably tight. Much of the between-organism variation in microbial mutation rate is associated with variation in genome size, in which larger genomes tend to have smaller per-base-pair mutation rates [28]. We account for this in our analysis of the data in Fig 1 by allowing mutation rates to vary among organisms and accounting for their phylogeny (explicitly including a typical genome size for each organism makes little difference to this analysis, Model S-I in S1 Text). However, the within-organism variation in mutation rate associated with *D* is strong enough to explain radical differences in mutation-rate estimates for the same organism within and between laboratories, without assuming any inconsistency in the fluctuation test itself. For instance, the estimates for *Salmonella* at the bottom of Fig 1 vary by over an order of magnitude but diverge little from a negatively sloping straight line. This suggests that, once population density is taken into account, fluctuation tests can give a more precise estimate of mutation rate than previously believed.

Fluctuation tests, however, have drawbacks, such as the possibility of unanticipated selection on mutant cells in a supposedly nonselective environment [19]. We avoided that specific issue here by using short incubation times and making estimates at multiple loci. The more general drawback of using fluctuation tests for considering environmental correlates (shared with most other methods for estimating mutation rates) is that they average across the time-varying environment of batch culture. Thus almost any environmental variable, including population density, has no fixed value and is itself associated with many other characteristics of the culture, both environmental and organismal (e.g., times spent in different phases of the culture cycle). Thus, while we have demonstrated an association of mutation rate with final population density *D* (Fig 2) and the dependence of that association on a downstream mechanism (Fig 4), the link between 8-oxo-dGTPase and *D* remains unclear. Nonetheless, 2 important things can be said about this link. First, DAMP is observed across a wide range of environmental conditions and organisms (Fig 1 and Fig 2), which argues that features of particular environments, such as the starting nutrient concentration, as manipulated here, are unlikely to provide a general link between *D* and mutation rate. Second, we have previously demonstrated that in 1 organism, *E*. *coli*, cell–cell interactions are involved in DAMP and deletion of a quorum-sensing gene (*luxS*) breaks the association between *D* and mutation rate [9], demonstrating that population interactions can be important for DAMP. Such quorum-sensing mechanisms occur widely (even in some phages [29]). Future work, therefore, needs to focus on asking whether DAMP is associated with particular environmental molecules.

Mutation rate is a central population genetic parameter. Across organisms, it is negatively associated with the other central population genetic parameter, effective population size (*N*_*e*_) [1]. In our experiments, despite 2 orders of magnitude variation in each measure, we find no consistent association between mutation rate and *N*_*e*_ (S9 Fig). This is unsurprising because the proposed reason for the negative association across organisms is that selection for replication fidelity is more efficient at higher *N*_*e*_, meaning that, over the long term, average mutation rates evolve to be lower at higher *N*_*e*_ [1]. In our short-term experiments, there is little opportunity for such evolutionary change to occur, so we do not see this association. Nonetheless, this reinforces the clear distinction between within-organism plasticity and among-organism variation in mutation rate. Both have shaped mutation rates in the published literature (Fig 1), and we are able to separate the 2, both statistically and by focused experiments (Fig 2). There may be links between the causes of among-organism variation and within-organism plasticity in mutation rate, for instance, in the differing opportunities for selection on replication fidelity in polymerases expressed in common or rare environmental conditions [30]. However, the evolutionary causes and effects of within-organism plasticity in mutation rate in general, and DAMP in particular, need further investigation.

The evolutionary causes of plasticity in mutation rates need not be adaptive [30]. Nonetheless, mutation is an evolutionary mechanism, so any plastic variation in mutation rates will have consequences for evolutionary trajectories [31]. What the evolutionary consequences might be depend on **how** mutation rate associates with the environment. For evolutionary computing, in which mutation rate is controlled, understanding the effect of that control is an important area of research [32]. In biology, constitutively high mutation rates can evolve under specific circumstances [33,34], but incur the costs of many, typically deleterious, mutations. If plasticity is such that mutation rate is inversely related to absolute organismal fitness, then organisms may benefit from a high mutation-supply rate without paying the full evolutionary cost of a constitutively raised mutation rate, as seen in mathematical studies of evolutionary systems [16] and population genetic models [35]. In some circumstances, DAMP can result in such a negative association of mutation rate with fitness [9], but the evolutionary effects of this remain to be tested.

The probability of a particular mutational event occurring (e.g., the emergence of spontaneous antibiotic resistance) might be expected to increase with *D*, as denser populations, containing more cells, will have had more opportunity for mutation. But for the DAMP described here, this increase is offset by a reduction in the mutation rate. This offsetting means that for organisms with DAMP, numbers of mutational events per space and time vary much less with *N*_*t*_ than expected from a fixed mutation rate per generation (S10 Fig). Population genetic models typically consider mutations per replication to be constant for an organism. However, we find that the approximate constant is the number of mutational events per space and time (S10 Fig). This is consistent with observations of invariant numbers of mutations per time in *Mycobacterium* infections [36] and, indeed, in human somatic [37] and germ cells [6].

Both the occurrence across domains of life (Fig 2) and the conserved mutation avoidance mechanism required (Fig 4) point to an ancient evolutionary origin for DAMP. Furthermore, Fig 1 suggests that DAMP also occurs in viruses and bacteriophage. Any variation in mutation rates in viruses and phage lacking mutation-avoidance or -correction mechanisms must be mediated by the host environment. Consistent with this, we see different mutation rates, but similar DAMP, for the same RNA virus in different host cells (S11 Fig reanalysed from [38]). DAMP itself, therefore, seems closely related to basic processes of replication common to all organisms. Nonetheless, our findings are limited to organisms in which it is possible to assay mutation rate by fluctuation tests. This excludes multicellular eukaryotes, so how our findings might apply to them is unclear. Recent findings of variation in mitochondrial mutation rates at different population sizes and densities of the nematode *Caenorhabditis elegans* highlight the challenge of separating out what population density could mean at the organism, tissue, cellular, and subcellular (e.g., mitochondrial) levels [39]. Even so, if it were possible to manipulate microbial DAMP clinically as well as genetically (Fig 4), for instance as a strategy to slow the rate at which antibiotic resistance arises [40], that could be applicable across the breadth of microbes, including pathogenic viruses.

## Materials and methods

### Strains used in this study

See S2 Table.

### Media

We used MilliQ water for all media. Tetrazolium arabinose agar (TA), Davis minimal medium (DM), and M9 minimal medium were prepared according to [41]. Luria-Bertani medium (LB), yeast extract peptone medium (YP), and yeast nitrogen base (YNB) were prepared according to manufacturers’ instructions. Magnesium sulphate heptahydrate, thiamine hydrochloride, carbon source (3 g/l L-arabinose or various concentrations of D-glucose), and 2,3,5-triphenyltetrazolium chloride (Sigma T8877) were sterile filtered and added to a cooled medium. Selective TA medium was supplemented with freshly prepared rifampicin (50 μg/ml) or nalidixic acid (30 μg/ml). Selective YP medium was supplemented with freshly prepared 5-FOA (1,000 μg/ml) or hygromycin B (300 μg/ml). For all cell dilutions, sterile saline (8.5 g/l NaCl) was used. All media were solidified as necessary with 15 g/l of agar (Difco).

### Fluctuation tests with bacteria

We did fluctuation tests with *E*. *coli* and *P*. *aeruginosa* as explained in [9]. In short, strains were first inoculated from frozen stock and grown in liquid LB medium at 37°C and then transferred to nonselective liquid DM (for *E*. *coli*) or M9 (for *P*. *aeruginosa*), supplemented with a particular concentration of glucose (25–300 mgl^−1^), and allowed to grow overnight shaking at 37°C. *E*. *coli* and *P*. *aeruginosa* were again diluted into fresh DM or M9 medium, respectively, giving the initial population size (*N*_*0*_) of around 10,000 (range 2.5×10^2^ to 1.3×10^5^) and 5,000 (range 2.5×10^3^ to 1.2×10^4^), respectively. Various volumes (0.5–10 ml) of parallel cultures were grown to saturation for 24 hours at 37°C in 96-deep-well plates or 50 ml falcon tubes. The position of each culture on a 96-well plate was chosen randomly. *N*_*t*_ of each culture was determined by 2 independent techniques. *N*_*t*_ was determined by CFU in which appropriate dilution was plated on a solid nonselective TA medium. Estimates of *N*_*t*_ using net luminescence were determined using a Promega GloMax luminometer and the Promega Bac-Titer Glo kit, according to manufacturer's instructions. We measured the luminescence of each culture 0.5 seconds and 510 seconds after adding the Bac-Titer Glo reagent and calculated net luminescence as LUM = LUM_510s_ − LUM_0.5s_. Each estimate of *N*_*t*_ is an average of 3 cultures. Evaporation (routinely monitored by weighing the plate before and after 24 hours of incubation) was accounted for in the *N*_*t*_ value determined by CFU and was also used in statistical modelling as a variance covariate. We obtained the observed number of mutants resistant to rifampicin or nalidixic acid, *r*, by plating the entirety of remaining cultures onto solid selective TA medium that allows spontaneous mutants to form colonies. Plates were incubated at 37°C, and mutants were counted at the earliest possible time after plating. For rifampicin plates, this was 44–48 hours, when nalidixic acid was used, the incubation time was 68–72 hours.

### Fluctuation tests with yeast

We did fluctuation tests with yeast in a similar way to fluctuation tests with bacteria (see above). Strains were inoculated from frozen stock in liquid YP medium with 20 mg/ml of glucose at 30°C (200 rpm) and then transferred to nonselective liquid YNB medium supplemented with a particular percentage of YP (*v*/*v*) and glucose, except for S288C in which YP was not added. We then allowed cultures to grow overnight at 30°C (200 rpm). Overnight cultures were again diluted into fresh medium giving *N*_*0*_ of around 5,000 per parallel culture (range 5×10^2^ to 5.1×10^4^). Various volumes of parallel cultures (0.35–10 ml) were grown in yeast nitrogen base with 25–8,000 mgl^−1^ glucose and 0%–7% *v*/*v* YP in 96-deep-well plates or in 50 ml falcon tubes to saturation for 48 or 72 hours at 30°C (200 rpm). We positioned each culture on the plate randomly. *N*_*t*_ was determined by CFU, in which an appropriate dilution was plated on solid nonselective YP medium. *N*_*t*_ determined with haemocytometer (Cellometer Auto M10 –Nexcelom) (CC) was done according to manufacturer’s instructions. *N*_*t*_ was calculated with 3 cultures per mutation-rate estimate, in which for each culture, CFU and CC were determined. Evaporation was accounted for in the *N*_*t*_ value determined by CFU and also used in statistical modelling as a variance covariate. We obtained the observed number of mutants resistant to 5-FOA or hygromycin B, *r*, by plating the entirety of remaining cultures onto solid selective YP medium. Plates were incubated at 30°C, and mutants were counted at the earliest possible time after plating, for both markers that was 68–72 hours.

For Figs 2A, 2B, 3, 4A, 4B, 4C and 4D, we used 21, 14, 14, 8, 5, 5, and 3 independent experimental blocks, respectively, carried out on different days. Within an experimental block, multiple 96-well plates, or groups of falcon tubes, were used. Any individual mutation-rate estimate requires multiple parallel cultures, which were all carried out on a particular plate, or group of falcon tubes. For Figs 2A, 2B, 3, 4A, 4B, 4C and 4D, the median number of parallel cultures used (with interquartile range) was 16 (15–16), 16 (16–16), 16 (16–16), 16 (16–16), 16 (16–16), 16 (16–16), and 16 (15–16), respectively.

### Estimation of mutation rates

To calculate *m* from the observed number of mutants, we employed the Ma-Sandri-Sarkar maximum-likelihood method implemented by the FALCOR web tool [42] or rSalvador [43,44]. The mutation rate per cell per generation is calculated as *m* divided by *N*_*t*_. The median (with interquartile range) of the coefficient of variation for *N*_*t*_ estimated with CFU and ATP-based luminescence assay is 15.9% (9.6%–24.8%) and 10.9% (6.9%–18.4%), respectively. *N*_*e*_ is calculated as the harmonic mean of the population size across generations.

### Statistical analysis

All statistical analysis was executed in R v3.2.4 and v3.3.1, respectively, when using spaMM v 1.7.2 (Model S-I, S1 Text) and nlme v3.1 (Model S-II to S-XVIII, S1 Text) packages for linear mixed effects modelling [45, 46]. This enabled the inclusion within the same model of experimental factors (fixed effects), blocking effects (random effects), and factors affecting variance (heteroscedasticity) as described in S1 Text. In all cases, the log_2_ mutation rate was used. Raw data (S1 Data, described in S4 Table) and R code (S1 Code) are provided, sufficient to reproduce Figs 1–4, S1, S3 and S4 Figs, S6 Fig and S8–S11 Figs.

### Whole genome sequencing

*E*. *coli* genomes were sequenced with the Illumina HiSeq2500 platform using 2 x 250 bp paired-end reads. Sequencing and initial read quality checking were provided by MicrobesNG (http://www.microbesng.uk) and deposited at the European Nucleotide Archive (accession number ERP024110, http://www.ebi.ac.uk/ena/data/view/ERP024110). Strains derived from MG1655 and from the Keio collection were aligned to the *E*. *coli* str. K-12 substr. MG1655 (NC_000913.3) and *E*. *coli* BW25113 (NZ_CP009273.1) genomes, respectively. Mutations (i.e., single nucleotide substitutions, small and large indels, and copy number variants) were predicted using breseq-0.27.2 using the default settings [47].

### Published mutation rate search criteria

We identified studies that used Luria-Delbrück fluctuation tests for estimating mutation rates. We considered all papers citing the original reference [10] and further searched the Google Scholar and Web of Science databases with keywords “mutation rate”, “Luria Delbruck”, “fluctuation test”, and “fluctuation assay”; we also considered papers cited by papers identified in this way. We collected mutation rate estimations from studies spanning over 70 years, starting with Luria and Delbrück’s pioneering paper in 1943 outlining the fluctuation test [10]. In all, we collected 474 mutation rate estimations from 68 separate studies, covering 26 different organisms from across domains of life (Archaea, Bacteria, Eukaryota) and viruses. From these studies, we recorded the mutation rate estimation, the estimator used for calculating mutation rate, the *D* of parallel cultures, identity of the nonselective medium, the organism studied, the selective marker used and its concentration, and the study the estimate came from. We excluded estimates that (i) involved microorganisms cultured in intentionally selective conditions, (ii) used genetically manipulated or mutator strains, or (iii) did not plate the entire culture volume onto the selective media. Any of this information that was not included in the published article was collected via a direct communication with the corresponding author.

Where the number of observed mutants per plate was available and the estimator was **not** the Ma-Sandri-Sarkar maximum-likelihood method, we recalculated the mutation rate using this method implemented by the FALCOR web tool [42]. When only the proportion of plates without mutants was available, we recalculated the mutation rate using the *P*_0_ method [14], implemented by equation −ln(*P*_0_)/*N*_*t*_ in which *P*_0_ is the proportion of plates containing no mutant colonies. For viral mutation rates, we recorded the mutation rate as substitutions per strand copying. Where the published mutation rates were not in this format, these were converted using equation 10 in [48]. All these points are highlighted in the column ‘recalculation’ as ‘yes’ in S1 Data (see S4 Table).

### Phylogeny used in analysing published mutation rates

To take account of the fact that organisms may show similarity (including in mutation rates) through common ancestry, we accounted for phylogenetic relatedness using a correlation matrix within our Model S-I (S1 Text). This matrix was derived from a phylogeny (S1 Fig) constructed from a combination of 3 published phylogenies. All the bacteria and archaea came from the ‘All-Species Living Tree’ Project (LTP) [49] version LTPs123, and this phylogeny was used to add other organisms. *S*. *cerevisiae* was taken from [50], and viruses were taken from [51]. Branch lengths for *S*. *cerevisiae* and the viruses were scaled to correspond to those in the LTP tree as follows. For *S*. *cerevisiae*, average branch lengths from the tips to the last common ancestor of the archaea *Halobacterium* and *Sulfolobus* were compared for the LTP and Lane and Darst [50] trees. The ratio between them was applied to the branch length of *S*. *cerevisiae* in Lane and Darst phylogeny [50] before adding it to the LTP tree at the branch point of the bacteria and archaea. For the viruses, 4 common tips from both trees (*P*. *aeruginosa*, *Thermus thermophilus*, *S*. *cerevisiae*, and *Halobacterium*) were selected, and the distance of each to a shared common ancestor (*P*. *aeruginosa* with *T*. *thermophilus* and *S*. *cerevisiae* with *Halobacterium*) were plotted against each other. A straight line was then fitted through the origin, and the gradient of that line was used to correct the branch length of the viruses in the Nasir and Caetano-Anollés tree [51] before being added to the combined LTP/*S*. *cerevisiae* tree at the branch point of the 3 domains.

Some organisms in our analysis were not present in these phylogenies. These were as follows: separate serovars of *Salmonella enterica* (serovars Typhimurium and Enteritidis), which we treated as subspecies of *S*. *enterica* (*indica* and *enterica*, present in the tree); Vesicular stomatitis virus and Measles virus, which were combined into their common order of Mononegavirales; and Bacteriophage ΦX174, which was positioned at Bacteriophage M13.

## Supporting information

S1 Fig Phylogeny used in analysing published mutation rates. Phylogeny used to control for relatedness in Model S-I (S1 Text) analysing data in Fig 1. See Materials and Methods for construction and usage. Raw data is available in S1 Code.
(TIF)

S2 Fig Slope values from Model S-I. Each point represents the estimate of the within-species slope of log_2_ (mutation rate) with log_2_ (population density) from mixed effect Model S-I (S1 Text), which includes a random effect of organism on slope. Each value therefore represent the best linear unbiased prediction (BLUP) for that organism. The vertical line represents the overall estimated slope, fitted as a fixed effect in Model S-I. Raw data is available in S1 Data.
(TIF)

S3 Fig Calibration curves for final population density measured by counting colony forming units (CFU), against luminescence, assayed with the BacTiter-Glo assay (arbitrary units—AU). Calibration curves shown are from Model S-IV (*N* = 368) for *E. coli* and *P*. *aeruginosa* strains used in Figs 2–4 and Model S-II, Model S-VII, Model S-VIII and Model S-X. See S1 Text for model details and S2 Table for strain details. Raw data is available in S1 Data.
(TIF)

S4 Fig Density-associated mutation-rate plasticity (DAMP) in bacteria and yeast. Data as in Fig 2 but using CFU to estimate both population density and mutation rate. (A) Mutation rates to rifampicin (triangles) and nalidixic acid (circles) resistance in *E*. *coli* MG1655 (dark blue; *N* = 77) and *P*. *aeruginosa* PAO1 (light blue; *N* = 40). Lines are from Model S-V in S1 Text; *t*_80_ = 14; *P* = 6.5×10^-23^ that *E*. *coli* slope is zero and *t*_80_ = 0.81, *P* = 0.42 that *P*. *aeruginosa* slope is zero (B) Mutation rates to hygromycin B (squares) and 5-FOA (diamonds) resistance in *S. cerevisiae* BY4742 (brown; *N* = 46), Sigma1278b (orange; *N* = 59) and S288C (red; *N* = 39). Lines are from Model S-VI in S1 Text: *t*_105_ = 4.3; *P* = 3.2×10^-5^ Wald test that average *S*. *cerevisiae* slope is zero. Open shapes denote mutation rate estimates which would typically be excluded because the estimated number of mutational events per culture, *m*, is either below 0.3 or above 30. Note the logarithmic axes. Raw data is available in S1 Data.
(TIF)

S5 Fig Effect of fitness differences between resistant and non-resistant strains on the estimated slopes in Fig 2. The estimated slope (with its standard error, grey ribbon) of mutation rate against population density, *D*, for *E*. *coli* (A) as shown in Fig 2A and estimated by Model S-II in S1 Text, having used different assumed average relative fitnesses (*w*) in the calculation of each mutation rate. The vertical red line indicates equal fitness of resistant and non-resistant strains, as assumed in the main analysis. Values of *w* below 1 indicate a cost of resistance and values greater than 1 indicate a selective advantage to resistant strains. The vertical dashed black lines indicate the 95% CI of fitness values *w* estimated directly from the data, jointly with the number of mutational events *m*. Because our fluctuation tests used relatively limited numbers of cultures (see methods), it was not possible to jointly estimate *m* and *w* from the data in all cases. The interval shown is the confidence interval on the mean across the *N* = 56 fluctuation tests where it was possible to make joint estimates. (B) The same as part A but for the slope of mutation rate against population density *D* for strain BY4742 as shown in Fig 2B and estimated by Model S-III (S1 Text); the confidence interval on the mean relative fitness of resistant strains was calculated across *N* = 84 fluctuation tests in this case. Raw data is available in S1 Data.
(TIF)

S6 Fig Relative fitness of rifampicin resistant mutants of *E. coli* REL606 (mutant A) and REL607 (mutant B) at different population densities. Rifampicin resistant mutant A and rifampicin resistant mutant B were competed against a rifampicin susceptible parent strain with the opposite arabinose marker (REL607 and REL606, respectively) in Davis minimal medium with 100 and 250 mgl^-1^ of glucose. Raw data is available in S1 Data.
(TIF)

S7 Fig All data from Fig 2 overlaid on published data used in Fig 1. Mutation rates in *E*. *coli* MG1655 (dark blue triangles), *P*. *aeruginosa* PAO1 (pale blue triangles) and *S*. *cerevisiae* (red squares) overlaid on published mutation rates collected from the literature (grey symbols). Green triangles represent mutation rate estimates for monocultures of wild-type *E*. *coli* from Krašovec *et al*. (2014) [9], which are not included in Fig 1. See main text and Fig 2 for more details. Raw data is available in S1 Data and [9].
(TIF)

S8 Fig DAMP in cells deficient in mutation avoidance or correction genes in *E*. *coli* and *S*. *cerevisiae*. Data as in Fig 4 but using alternative methods to estimate population density (A) Mutation rates to nalidixic acid resistance in two independent *E*. *coli* Keio Δ*mutT* strains JW0097-1 (*N* = 30) and JW0097-3 (*N* = 33) (dark and light blue respectively). Both lines result from a Model S-IX in S1 Text; likelihood ratio test of slope, *N* = 63, *LR*_1_ = 0.29; *P* = 0.59 (B) Mutation rates to rifampicin resistance in *E*. *coli* Δ*mutM* (*N* = 23) and Δ*mutY* (*N* = 23) strains (blue and green respectively). Line result from a Model S-XI in S1 Text. Wald tests that slope is zero for Δ*mutM t*_32_ = 12.9; *P* = 3.5×10^-14^ and ΔmutY *t*_32_ = 9.8; *P* = 3.3×10^-11^ (C) Mutation rate to hygromycin B resistance in *S*. *cerevisiae* BY4742 (*N* = 22) and Sigma1278b *PCD1*-Δ (*N* = 20) strains (brown and orange respectively). Line result from a Model S-XIII in S1 Text (D) Mutation rate to 5-FOA resistance in *S*. *cerevisiae Sigma1278b MLH1*-Δ. Line results from Model S-XV in S1 Text. Wald test that the slope is zero: *N* = 14, *t*_10_ = 3.5; *P* = 0.0035. Final Density (*D*) measured by CFU in (A) and (B), and by direct cell counts in (C) and (D). Open shapes denote mutation rate estimates which would typically be excluded because the estimated number of mutational events per culture, *m*, is either below 0.3 or above 30. Raw data is available in S1 Data.
(TIF)

S9 Fig Mutation rate in relation to *N_e_* for all genotypes tested. All mutation rates determined in this study are shown in relation to *N_e_*, which is calculated as the harmonic mean across generations of the population size as it increases from *N_0_* to *N_t_*. The plotted lines come from Model S-XVI in S1 Text (*N* = 580), and the data are separated into panels, primarily for clarity, according to the degree of mutation rate plasticity identified in Figs 2–4. Raw data is available in S1 Data.
(TIFF)

S10 Fig Number of mutational events *m* per space and time in response to final population size *N_t_*for all genotypes tested. The estimated number of mutational events, *m*, is elsewhere divided through by *N_t_* to give the mutation rate per generation. Here all mutation rates determined in this study are plotted against *N_t_*, having divided through by both the culture time in hours (typically 24h) and the culture volume in ml. The black dashed line indicates a slope of 1 (doubling *N_t_*is associated with doubling *m* for a given volume and time), which is the expectation for a fixed (non-plastic) mutation rate. The colored lines come from Model S-XVII in S1 Text (*N* = 580), and the data are separated into panels, primarily for clarity, according to the degree of mutation rate plasticity identified in Figs 2–4. Raw data is available in S1 Data.
(TIF)

S11 Fig Density-associated mutation-rate plasticity (DAMP) in Vesicular stomatitis virus hosted by different cell lines. Data from Sanjuan *et al*. (2010) [47], plaque forming units was used to estimate population density of viral particles. Viral mutation rates to monoclonal antibody resistance were estimated in different host cells grown in normal (21%) oxygen levels at 37°C (squares) or 28°C (triangles) and in low (1%) oxygen levels at 37°C (circles). Hosts were baby hamster kidney cells (BHK), CT26 colon cancer cells (CT26), wild-type and Δ*p53* primary mouse embryonic fibroblasts (MEF and MEF_p53, respectively), Neuro-2a neuroblastoma cells (Neuro), ovarian cells of the moth (sf21) and that of mosquito larvae (C6/36). Lines are from Model S-XVIII in S1 Text (*N* = 34, likelihood ratio test that host environment has no effect on the viral mutation rate *LR*_7_ = 68, *P* = 4.3×10^-12^). Note the logarithmic axes. Raw data is available in S1 Data.
(TIF)

S1 Table List of papers from which mutation rate estimates in Fig 1 are taken. (DOCX)

S2 Table Bacterial and yeast strains. (DOCX)

S3 Table Breseq analysis of mutations identified in genome sequence for two Δ*mutT *Keio strains. See Materials and Methods for more details about the analysis. Differences from the reference common to all Keio strains are not shown. Sequence data available at the European Nucleotide Archive (accession number ERP024110, http://www.ebi.ac.uk/ena/data/view/ERP024110).
(DOCX)

S4 Table Detailed description of the columns in the raw data file S1 Data. (DOCX)

S1 Text Statistical models. Descriptions of all statistical models, including ANOVA tables and diagnostic plots.
(DOCX)

S1 Data Raw data used in this study. Data underlying Figs 1–4 and S2–S11 Figs. File may be directly used with S1 Code and the R language, to reproduce Figs 1–4 and S3, S4, S6 and S8–S11 Figs.
(CSV)

S1 Code R code to reproduce Figs 1–4. Sufficient, with S1 Data and the R language, to reproduce Figs 1–4, S1, S3, S4, S6 and S8–S11 Figs.
(R) Click here for additional data file.

## Abbreviations

5-FOA: 5-Fluoro-orotic acid
CC: Cell Counts
CFU: Colony-Forming Units
*D*: The final population density
DAMP: Density-associated mutation-rate plasticity
GO: 8-Hydroxyguanine
m: Number of mutational events
MMR: Methyl-directed DNA mismatch repair
*N_0_*: The initial population size
*N_t_*: The final population size
*N_e_*: The effective population size
