# Supplementary figures and images for: Spontaneous mutation rate is a plastic trait associated with population density across domains of life
Source: PLoS Biol. 2017 Aug 24;15(8):e2002731. doi: 10.1371/journal.pbio.2002731 (PMC5570273; doi:10.1371/journal.pbio.2002731)

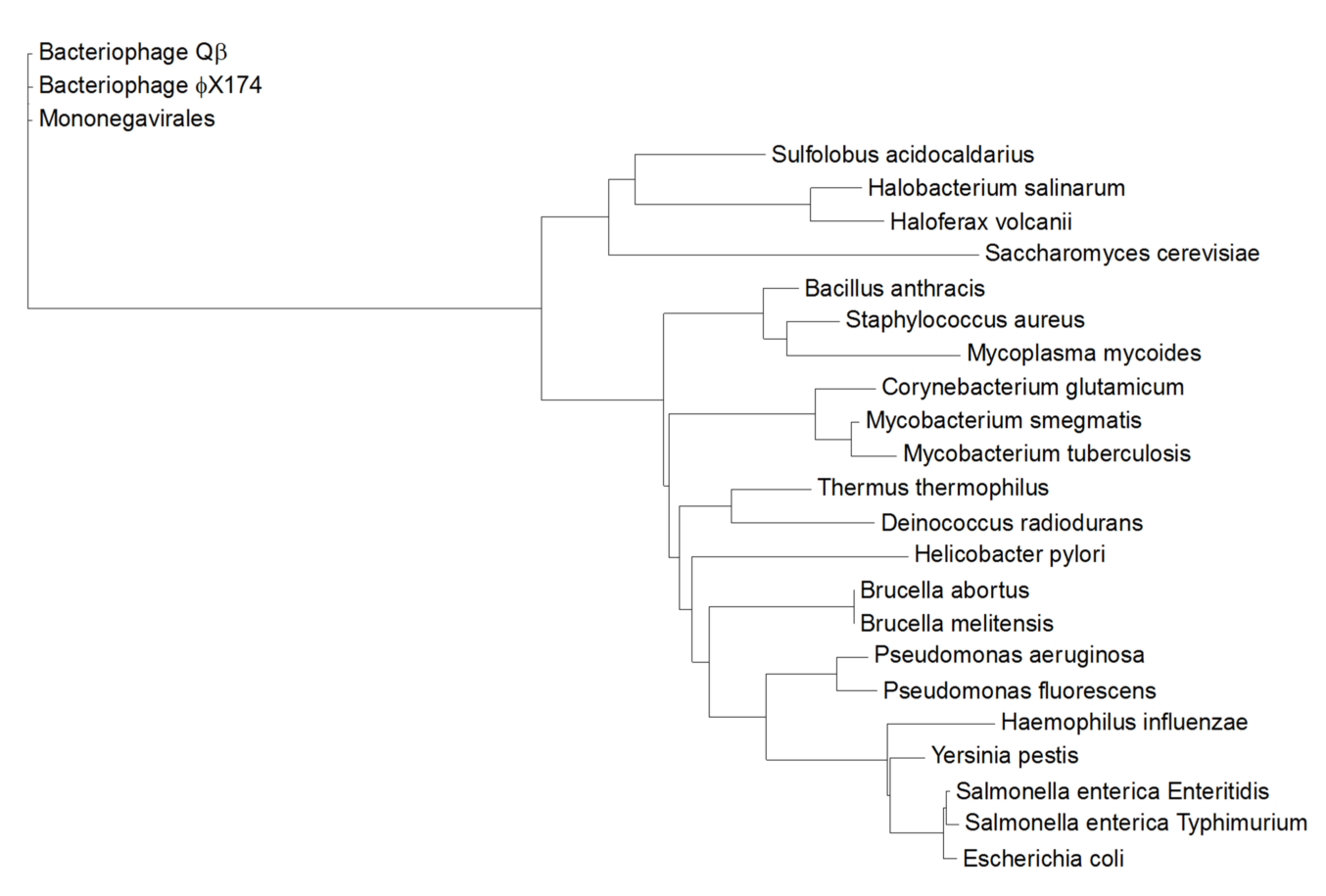

Supplement: S1 Fig — Phylogeny used to control for relatedness in Model S-I (S1 Text) analysing data in Fig 1. See Materials and Methods for construction and usage. Raw data is available in S1 Code. (TIF) [file pbio.2002731.s001.tif]

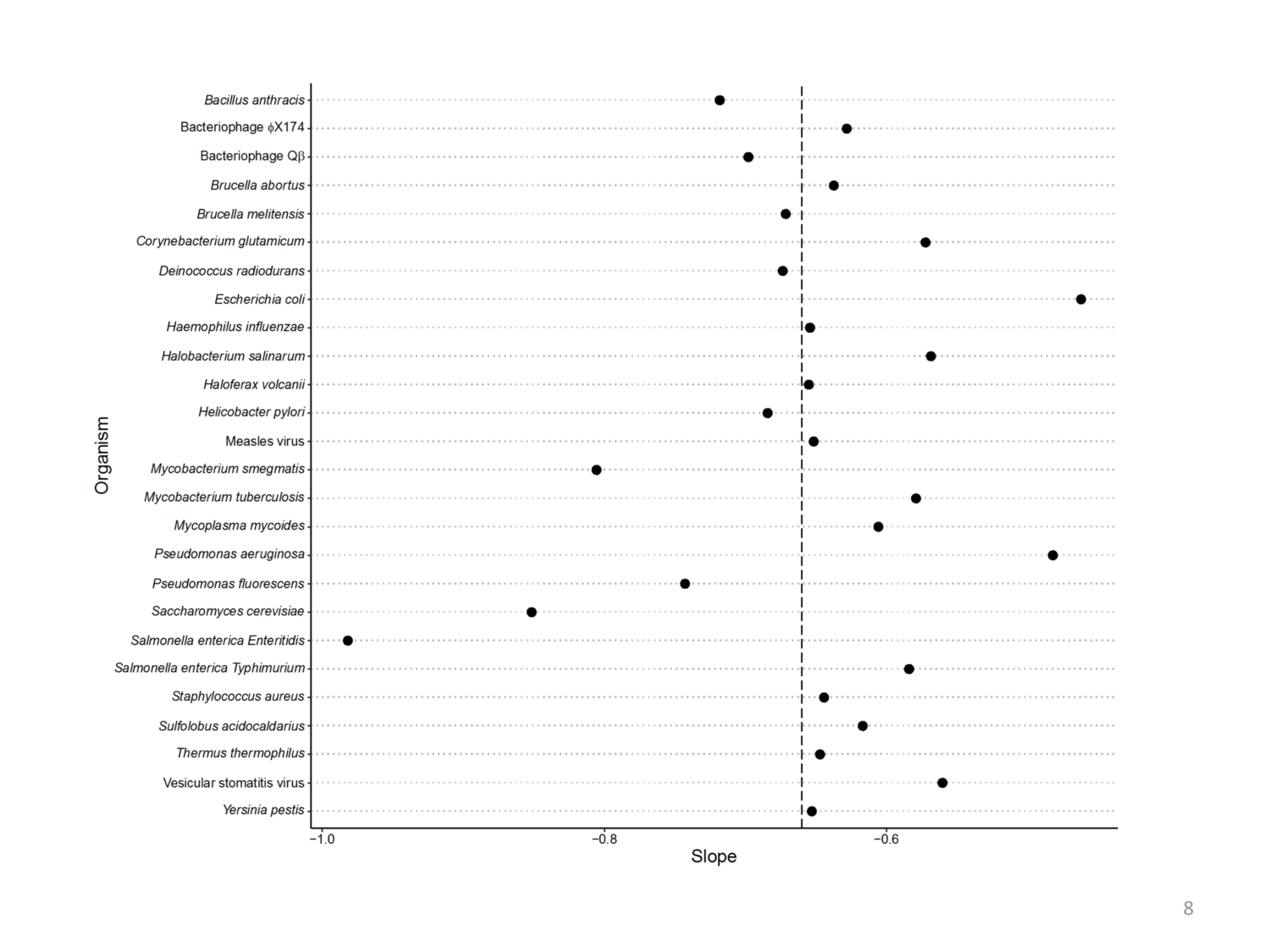

Supplement: S2 Fig — Each point represents the estimate of the within-species slope of log2 (mutation rate) with log2 (population density) from mixed effect Model S-I (S1 Text), which includes a random effect of organism on slope. Each value therefore represent the best linear unbiased prediction (BLUP) for that organism. The vertical line represents the overall estimated slope, fitted as a fixed effect in Model S-I. Raw data is available in S1 Data. (TIF) [file pbio.2002731.s002.tif]

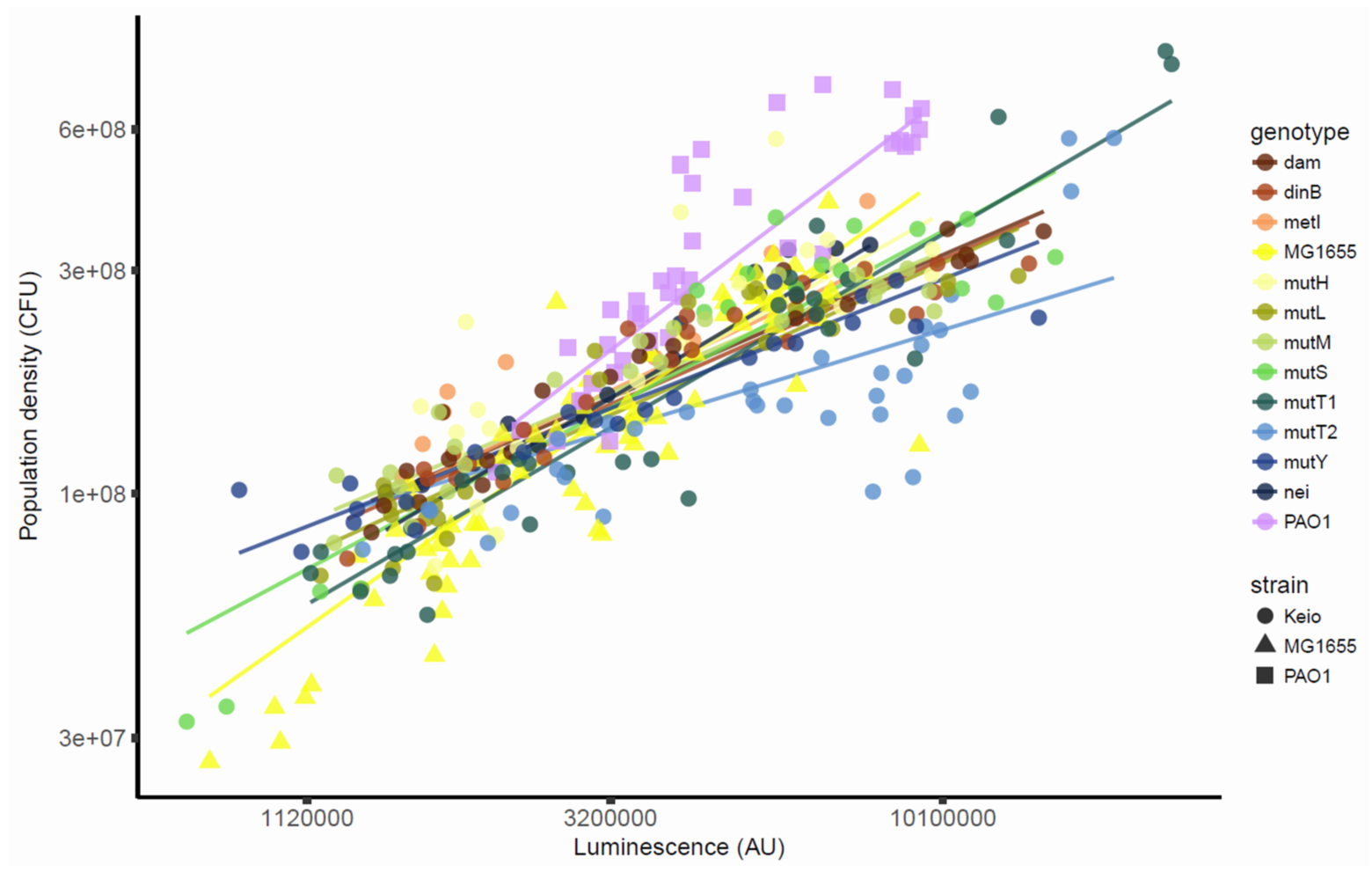

Supplement: S3 Fig — Calibration curves shown are from Model S-IV (N = 368) for E. coli and P. aeruginosa strains used in Figs 2–4 and Model S-II, Model S-VII, Model S-VIII and Model S-X. See S1 Text for model details and S2 Table for strain details. Raw data is available in S1 Data. (TIF) [file pbio.2002731.s003.tif]

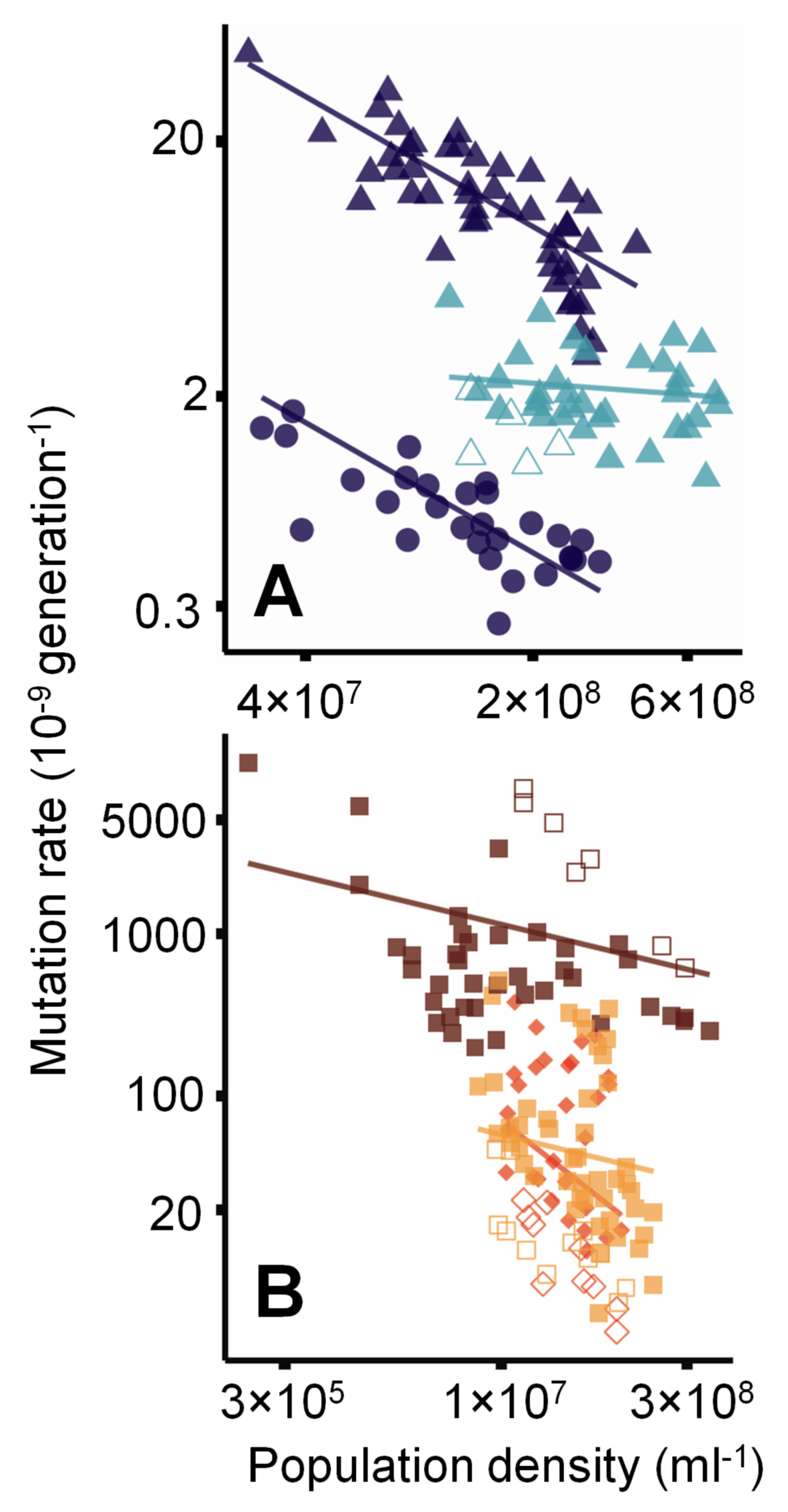

Supplement: S4 Fig — Data as in Fig 2 but using CFU to estimate both population density and mutation rate. (A) Mutation rates to rifampicin (triangles) and nalidixic acid (circles) resistance in E. coli MG1655 (dark blue; N = 77) and P. aeruginosa PAO1 (light blue; N = 40). Lines are from Model S-V in S1 Text; t80 = 14; P = 6.5×10^-23^ that E. coli slope is zero and t80 = 0.81, P = 0.42 that P. aeruginosa slope is zero (B) Mutation rates to hygromycin B (squares) and 5-FOA (diamonds) resistance in S. cerevisiae BY4742 (brown; N = 46), Sigma1278b (orange; N = 59) and S288C (red; N = 39). Lines are from Model S-VI in S1 Text: t105 = 4.3; P = 3.2×10^-5^ Wald test that average S. cerevisiae slope is zero. Open shapes denote mutation rate estimates which would typically be excluded because the estimated number of mutational events per culture, m, is either below 0.3 or above 30. Note the logarithmic axes. Raw data is available in S1 Data. (TIF) [file pbio.2002731.s004.tif]

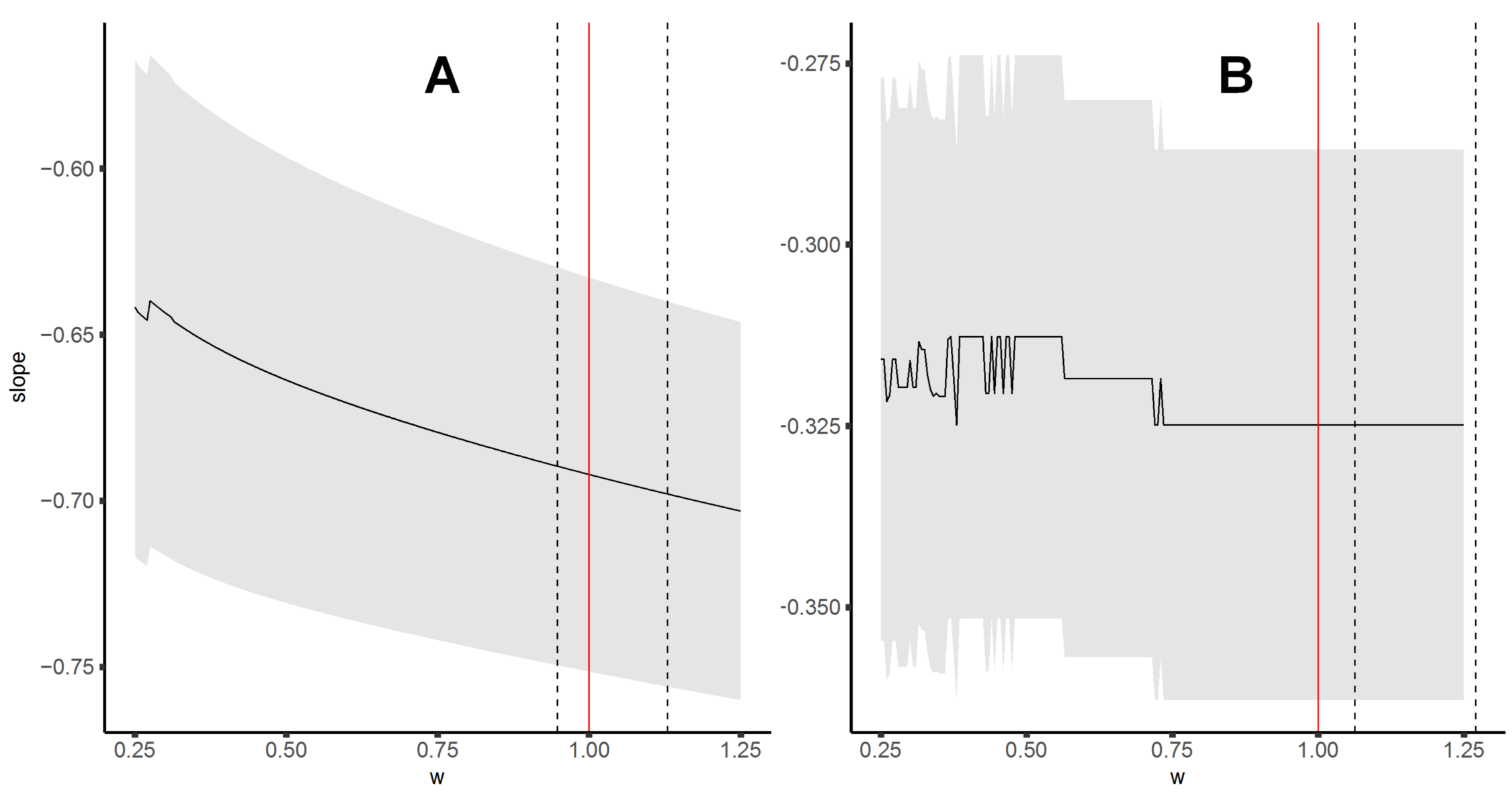

Supplement: S5 Fig — The estimated slope (with its standard error, grey ribbon) of mutation rate against population density, D, for E. coli (A) as shown in Fig 2A and estimated by Model S-II in S1 Text, having used different assumed average relative fitnesses (w) in the calculation of each mutation rate. The vertical red line indicates equal fitness of resistant and non-resistant strains, as assumed in the main analysis. Values of w below 1 indicate a cost of resistance and values greater than 1 indicate a selective advantage to resistant strains. The vertical dashed black lines indicate the 95% CI of fitness values w estimated directly from the data, jointly with the number of mutational events m. Because our fluctuation tests used relatively limited numbers of cultures (see methods), it was not possible to jointly estimate m and w from the data in all cases. The interval shown is the confidence interval on the mean across the N = 56 fluctuation tests where it was possible to make joint estimates. (B) The same as part A but for the slope of mutation rate against population density D for strain BY4742 as shown in Fig 2B and estimated by Model S-III (S1 Text); the confidence interval on the mean relative fitness of resistant strains was calculated across N = 84 fluctuation tests in this case. Raw data is available in S1 Data. (TIF) [file pbio.2002731.s005.tif]

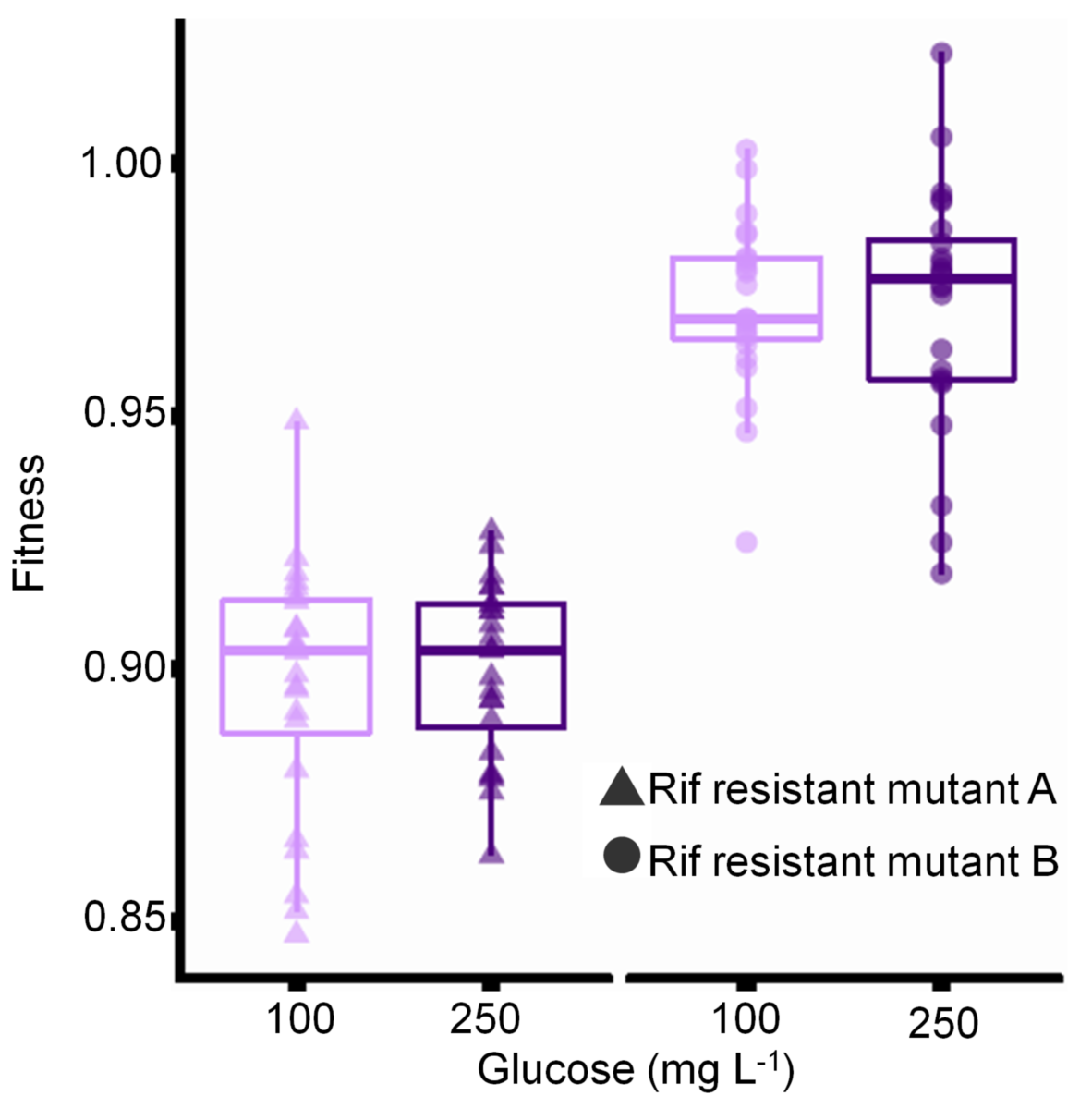

Supplement: S6 Fig — Rifampicin resistant mutant A and rifampicin resistant mutant B were competed against a rifampicin susceptible parent strain with the opposite arabinose marker (REL607 and REL606, respectively) in Davis minimal medium with 100 and 250 mgl-1 of glucose. Raw data is available in S1 Data. (TIF) [file pbio.2002731.s006.tif]

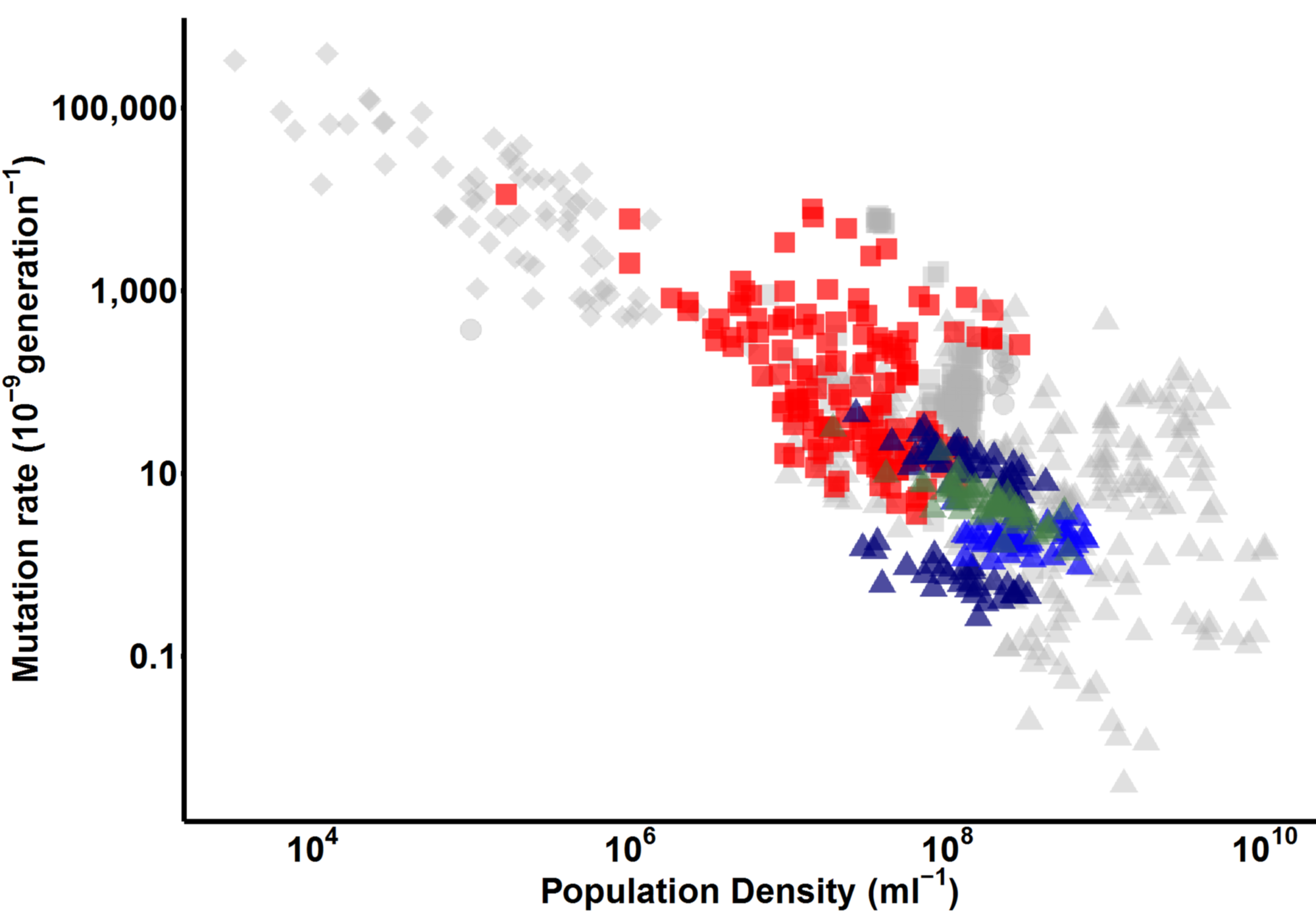

Supplement: S7 Fig — Mutation rates in E. coli MG1655 (dark blue triangles), P. aeruginosa PAO1 (pale blue triangles) and S. cerevisiae (red squares) overlaid on published mutation rates collected from the literature (grey symbols). Green triangles represent mutation rate estimates for monocultures of wild-type E. coli from Krašovec et al. (2014) [9], which are not included in Fig 1. See main text and Fig 2 for more details. Raw data is available in S1 Data and [9]. (TIF) [file pbio.2002731.s007.tif]

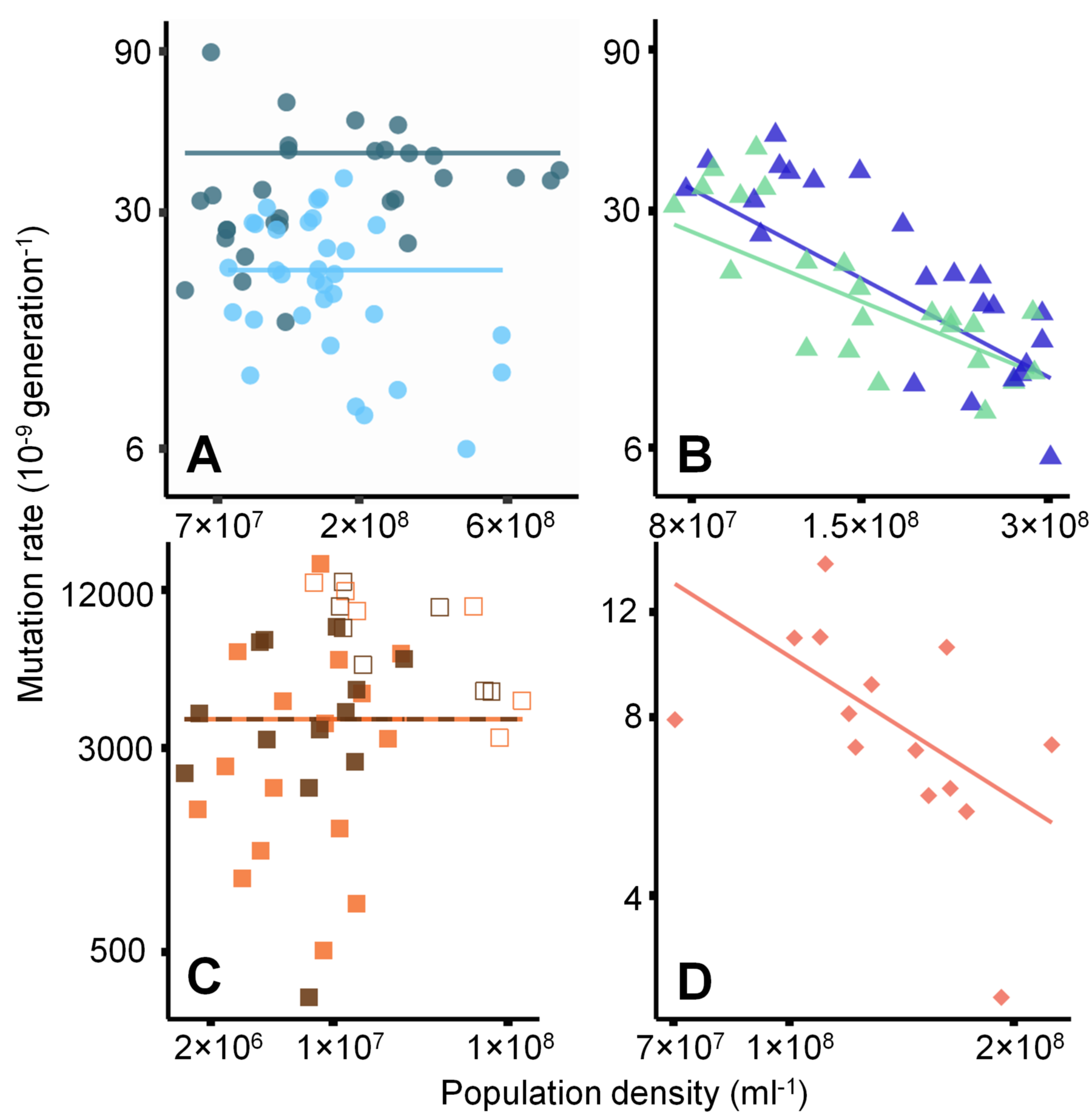

Supplement: S8 Fig — Data as in Fig 4 but using alternative methods to estimate population density (A) Mutation rates to nalidixic acid resistance in two independent E. coli Keio ΔmutT strains JW0097-1 (N = 30) and JW0097-3 (N = 33) (dark and light blue respectively). Both lines result from a Model S-IX in S1 Text; likelihood ratio test of slope, N = 63, LR1 = 0.29; P = 0.59 (B) Mutation rates to rifampicin resistance in E. coli ΔmutM (N = 23) and ΔmutY (N = 23) strains (blue and green respectively). Line result from a Model S-XI in S1 Text. Wald tests that slope is zero for ΔmutM t32 = 12.9; P = 3.5×10^-14^ and ΔmutY t32 = 9.8; P = 3.3×10^-11^ (C) Mutation rate to hygromycin B resistance in S. cerevisiae BY4742 (N = 22) and Sigma1278b PCD1-Δ (N = 20) strains (brown and orange respectively). Line result from a Model S-XIII in S1 Text (D) Mutation rate to 5-FOA resistance in S. cerevisiae Sigma1278b MLH1-Δ. Line results from Model S-XV in S1 Text. Wald test that the slope is zero: N = 14, t10 = 3.5; P = 0.0035. Final Density (D) measured by CFU in (A) and (B), and by direct cell counts in (C) and (D). Open shapes denote mutation rate estimates which would typically be excluded because the estimated number of mutational events per culture, m, is either below 0.3 or above 30. Raw data is available in S1 Data. (TIF) [file pbio.2002731.s008.tif]

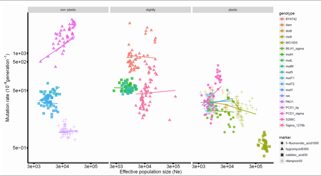

Supplement: S9 Fig — All mutation rates determined in this study are shown in relation to Ne, which is calculated as the harmonic mean across generations of the population size as it increases from N0 to Nt. The plotted lines come from Model S-XVI in S1 Text (N = 580), and the data are separated into panels, primarily for clarity, according to the degree of mutation rate plasticity identified in Figs 2–4. Raw data is available in S1 Data. (TIFF) [file pbio.2002731.s009.tiff]

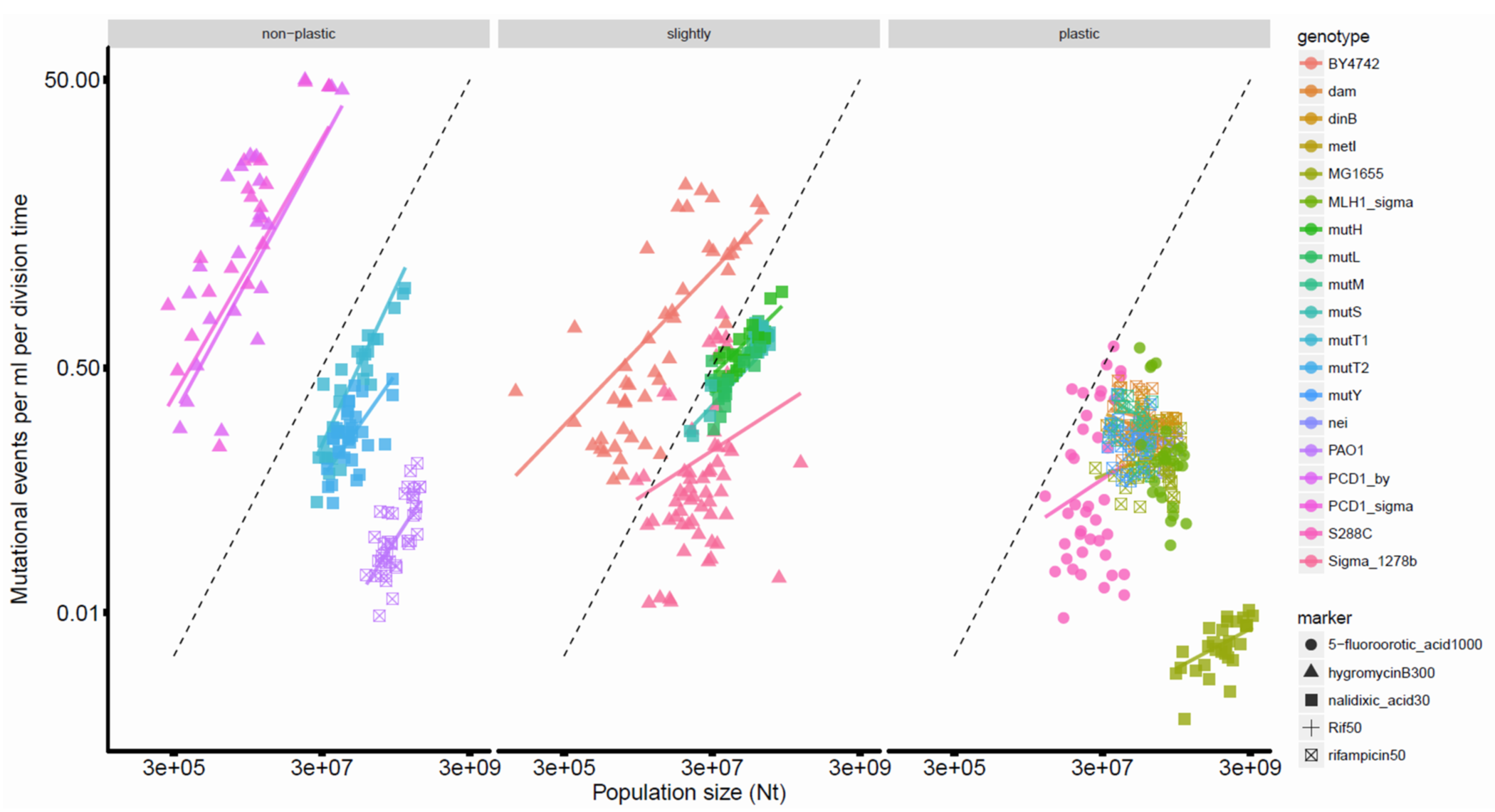

Supplement: S10 Fig — The estimated number of mutational events, m, is elsewhere divided through by Nt to give the mutation rate per generation. Here all mutation rates determined in this study are plotted against Nt, having divided through by both the culture time in hours (typically 24h) and the culture volume in ml. The black dashed line indicates a slope of 1 (doubling Ntis associated with doubling m for a given volume and time), which is the expectation for a fixed (non-plastic) mutation rate. The colored lines come from Model S-XVII in S1 Text (N = 580), and the data are separated into panels, primarily for clarity, according to the degree of mutation rate plasticity identified in Figs 2–4. Raw data is available in S1 Data. (TIF) [file pbio.2002731.s010.tif]

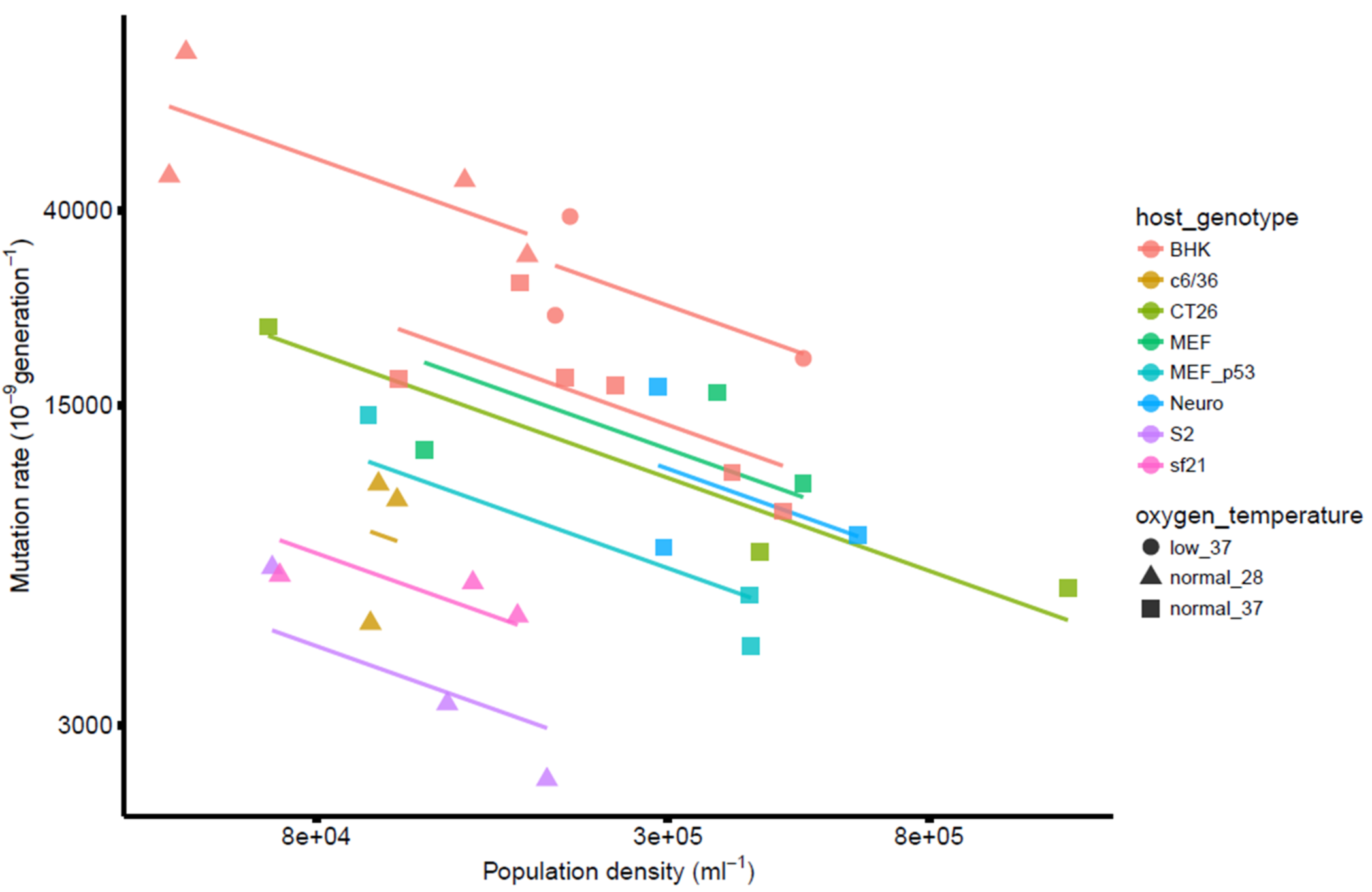

Supplement: S11 Fig — Data from Sanjuan et al. (2010) [47], plaque forming units was used to estimate population density of viral particles. Viral mutation rates to monoclonal antibody resistance were estimated in different host cells grown in normal (21%) oxygen levels at 37°C (squares) or 28°C (triangles) and in low (1%) oxygen levels at 37°C (circles). Hosts were baby hamster kidney cells (BHK), CT26 colon cancer cells (CT26), wild-type and Δp53 primary mouse embryonic fibroblasts (MEF and MEF_p53, respectively), Neuro-2a neuroblastoma cells (Neuro), ovarian cells of the moth (sf21) and that of mosquito larvae (C6/36). Lines are from Model S-XVIII in S1 Text (N = 34, likelihood ratio test that host environment has no effect on the viral mutation rate LR7 = 68, P = 4.3×10^-12^). Note the logarithmic axes. Raw data is available in S1 Data. (TIF) [file pbio.2002731.s011.tif]
