# Supplementary material for: Spontaneous mutation rate is a plastic trait associated with population density across domains of life
Source: PLoS Biol. 2017 Aug 24;15(8):e2002731. doi: 10.1371/journal.pbio.2002731 (PMC5570273; doi:10.1371/journal.pbio.2002731)
Supplement: S1 Table — (DOCX) [file pbio.2002731.s012.docx]

S1 Table**. List of papers from which mutation rate estimates in Fig 1 are taken.**

| 1. | C. Aguilar *et al.*, Deletion of the 2-acyl-glycerophosphoethanolamine cycle improve glucose metabolism in *Escherichia coli* strains employed for overproduction of aromatic compounds. *Microb. Cell. Fact.* **14**, 194 (2015). |
| --- | --- |
| 2. | F. I. Arias-Sánchez, A. R. Hall, Effects of antibiotic resistance alleles on bacterial evolutionary responses to viral parasites. *Biol. Lett.* **12**, 20160064 (2016). |
| 3. | L. Boe, Translational errors as the cause of mutations in *Escherichia coil*. *Mol. Gen. Genet.* **231**, 469-471 (1992). |
| 4. | H. Boshoff, M. Reed, C. B. III, V. Mizrahi, DnaE2 Polymerase Contributes to In Vivo Survival and the Emergence of Drug Resistance in *Mycobacterium tuberculosis*. *Cell* **113**, 183-193 (2003). |
| 5. | K. Bradwell, M. Combe, P. Domingo-Calap, R. Sanjuán, Correlation between mutation rate and genome size in riboviruses: Mutation rate of bacteriophage Qβ. *Genetics* **195**, 243-251 (2013). |
| 6. | S. Broomfield, B. L. Chow, W. Xiao, MMS2, encoding a ubiquitin-conjugating-enzyme-like protein, is a member of the yeast error-free postreplication repair pathway. *Proc. Natl. Acad. Sci. U. S. A.* **95**, 5678-5683 (1998). |
| 7. | C. R. Busch, J. DiRuggiero, MutS and MutL are dispensable for maintenance of the genomic mutation rate in the halophilic archaeon *Halobacterium salinarum* NRC-1. *PLoS ONE* **5**, 1-8 (2010). |
| 8. | M. Combe, R. Sanjuán, Variation in RNA Virus Mutation Rates across Host Cells. *PLoS Pathogens* **10**, (2014). |
| 9. | B. Csörgo, T. Fehér, E. Tímár, F. R. Blattner, G. Pósfai, Low-mutation-rate, reduced-genome *Escherichia coli*: an improved host for faithful maintenance of engineered genetic constructs. *Microb. Cell. Fact.* **11**, 11 (2012). |
| 10. | J. M. Cuevas, S. Duffy, R. Sanjuán, Point mutation rate of bacteriophage ΦX174. *Genetics* **183**, 747-749 (2009). |
| 11. | H. L. David, Probability Distribution of Drug-Resistant Mutants in Unselected Populations of *Mycobacterium tuberculosis*. *Appl. Envir. Microbiol.* **20**, 810-814 (1970). |
| 12. | M. Demerec, Studies of the Streptomycin-Resistance System of Mutations in *E. Coli.* *Genetics* **36**, 585-597 (1951). |
| 13. | M. Demerec, U. Fano, Bacteriophage-Resistant Mutants in *Escherichia coli*. *Genetics* **30**, 119 (1945). |
| 14. | P. Domingo-Calap, M. Pereira-Gómez, R. Sanjuán, Nucleoside analogue mutagenesis of a single-stranded DNA virus: evolution and resistance. *J. Virol.* **86**, 9640-9646 (2012). |
| 15. | M. S. Esposito, C. V. Bruschi, Diploid yeast cells yield homozygous spontaneous mutations. *Curr. Genet.* **23**, 430-434 (1993). |
| 16. | M. S. Esposito, R. M. Ramirez, C. V. Bruschi, Nonrandomly-associated forward mutation and mitotic recombination yield yeast diploids homozygous for recessive mutations. *Curr. Genet.* **26**, 302-307 (1994). |
| 17. | T. Feher, B. Cseh, K. Umenhoffer, I. Karcagi, G. Posfai, Characterization of *cycA* mutants of *Escherichia coli*. An assay for measuring in vivo mutation rates. *Mutat. Res.* **595**, 184-190 (2006). |
| 18. | C. B. Ford *et al.*, Use of whole genome sequencing to estimate the mutation rate of *Mycobacterium tuberculosis* during latent infection. *Nat. Genet.* **43**, 482-486 (2011). |
| 19. | C. B. Ford *et al.*, Emergence of Drug Resistant Tuberculosis. *Nat. Genet.* **45**, 784-790 (2013). |
| 20. | V. Furió, A. Moya, R. Sanjuán, The cost of replication fidelity in an RNA virus. *Proc. Natl. Acad. Sci. U. S. A.* **102**, 10233-10237 (2005). |
| 21. | W. E. Glaab, L. S. Mitchell, J. E. Miller, K. Vlasakova, T. R. Skopek, 5-Fluorouracil forward mutation assay in *Salmonella*: Determination of mutational target and spontaneous mutational spectra. *Mutat. Res.* **578**, 238-246 (2005). |
| 22. | B. Grimberg, C. Zeyl, the Effects of Sex and Mutation Rate on Adaptation in Test Tubes. *Evolution* **59**, 431-438 (2005). |
| 23. | B. G. Hall, Activation of the bgl operon by adaptive mutation. *Mol. Biol. Evol.* **15**, 1-5 (1998). |
| 24. | F. Hassim, A. O. Papadopoulos, B. D. Kana, B. G. Gordhan, A combinatorial role for MutY and Fpg DNA glycosylases in mutation avoidance in *Mycobacterium smegmatis*. *Mutation Research - Fundamental and Molecular Mechanisms of Mutagenesis* **779**, 24-32 (2015). |
| 25. | E. Huitric *et al.*, Rates and mechanisms of resistance development in *Mycobacterium tuberculosis* to a novel diarylquinoline ATP synthase inhibitor. *Antimicrob. Agents Chemother.* **54**, 1022-1028 (2010). |
| 26. | K. L. Jacobs, D. W. Grogan, Rates of spontaneous mutation in an archaeon from geothermal environments. *J. Bacteriol.* **179**, 3298-3303 (1997). |
| 27. | S. M. Karve *et al.*, *Escherichia coli* populations in unpredictably fluctuating environments evolve to face novel stresses through enhanced efflux activity. *J. Evol. Biol.* **28**, 1131-1143 (2015). |
| 28. | P. Komp Lindgren, A. Karlsson, D. Hughes, Mutation rate and evolution of fluoroquinolone resistance in *Escherichia coli* isolates from patients with urinary tract infections. *Antimicrob. Agents Chemother.* **47**, 3222-3232 (2003). |
| 29. | K. Kurthkoti *et al.*, A distinct physiological role of MutY in mutation prevention in mycobacteria. *Microbiology* **156**, 88-98 (2010). |
| 30. | K. Kuthkoti, P. Kumar, R. Jain, U. Varshney, Important role of the nucleotide excision repair pathway in *Mycobacterium smegmatis* in conferring protection against commonly encountered DNA-damaging agents. *Microbiology* **154**, 2776-2785 (2008). |
| 31. | G. I. Lang, A. W. Murray, Estimating the per-base-pair mutation rate in the yeast *Saccharomyces cerevisiae*. *Genetics* **178**, 67-82 (2008). |
| 32. | G. I. Lang, A. W. Murray, Mutation rates across budding yeast chromosome VI Are correlated with replication timing. *Genome Biol. Evol.* **3**, 799-811 (2011). |
| 33. | D. H. Lee, R. J. Miles, J. R. Inal, Antibiotic sensitivity and mutation rates to antibiotic resistance in *Mycoplasma mycoides* ssp. *mycoides*. *Epidemiol. Infect.* **98**, 361-368 (1987). |
| 34. | R. E. Lenski *et al.*, Sustained fitness gains and variability in fitness trajectories in the long-term evolution experiment with Escherichia coli. *Proc Biol Sci* **282**, 20152292 (2015). |
| 35. | S. F. Levy *et al.*, Quantitative evolutionary dynamics using high-resolution lineage tracking. *Nature* **519**, 181-186 (2015). |
| 36. | H. Long *et al.*, Background mutational features of the radiation-resistant bacterium *Deinococcus radiodurans*. *Mol. Biol. Evol.* **32**, 2383-2392 (2015). |
| 37. | H. Long *et al.*, Mutation rate, spectrum, topology, and context-dependency in the DNA mismatch repair-deficient *Pseudomonas fluorescens* ATCC948. *Genome Biol. Evol.* **7**, 262-271 (2014). |
| 38. | S. E. Luria, M. Delbrück, Mutations of bacteria from virus sensitivity to virus resistance. *Genetics* **28**, 491-511 (1943). |
| 39. | E. E. Machowski, S. Barichievy, B. Springer, S. I. Durbach, V. Mizrahi, In vitro analysis of rates and spectra of mutations in a polymorphic region of the Rv0746 PE_PGRS gene of *Mycobacterium tuberculosis*. *J. Bacteriol.* **189**, 2190-2195 (2007). |
| 40. | M. D. Maciá *et al.*, Efficacy and potential for resistance selection of antipseudomonal treatments in a mouse model of lung infection by hypermutable *Pseudomonas aeruginosa*. *Antimicrob. Agents Chemother.* **50**, 975-983 (2006). |
| 41. | R. R. Mackwan, G. T. Carver, J. W. Drake, D. W. Grogan, An unusual pattern of spontaneous mutations recovered in the halophilic archaeon *Haloferax volcanii*. *Genetics* **176**, 697-702 (2007). |
| 42. | R. R. Mackwan, G. T. Carver, G. E. Kissling, J. W. Drake, D. W. Grogan, The rate and character of spontaneous mutation in *Thermus thermophilus*. *Genetics* **180**, 17-25 (2008). |
| 43. | V. S. Malshetty, R. Jain, T. Srinath, K. Kurthkoti, U. Varshney, Synergistic effects of UdgB and Ung in mutation prevention and protection against commonly encountered DNA damaging agents in *Mycobacterium smegmatis*. *Microbiology* **156**, 940-949 (2010). |
| 44. | A. E. Minias, A. M. Brzostek, P. Minias, J. Dziadek, The deletion of *rnhB* in *Mycobacterium smegmatis* does not affect the level of RNase HII substrates or influence genome stability. *PLoS ONE* **10**, e0115521 (2015). |
| 45. | M. R. Monti, V. Miguel, M. V. Borgogno, C. E. Argaraña, Functional analysis of the interaction between the mismatch repair protein MutS and the replication processivity factor β clamp in *Pseudomonas aeruginosa*. *DNA Repair* **11**, 463-469 (2012). |
| 46. | H. B. Newcombe, Delayed Phenotypic Expression of Spontaneous Mutations in *Escherichia Coli*. *Genetics* **33**, 447-476 (1948). |
| 47. | H. B. Newcombe, R. Hawirko, Spontaneous Mutation to Streptomycin Resistance and Dependence in *Escherichia coli*. *J. Bacteriol.* **57**, 565-572 (1949). |
| 48. | H. B. Newcombe, G. J. Mc, On the nonadaptive nature of change to full streptomycin resistance in *Escherichia coli*. *J. Bacteriol.* **62**, 539-544 (1951). |
| 49. | S. Oide *et al.*, Thermal and solvent stress cross-tolerance conferred to *Corynebacterium glutamicum* by adaptive laboratory evolution. *Appl. Environ. Microbiol.* **81**, 2284-2298 (2015). |
| 50. | M. Pereira-Gómez, R. Sanjuán, Delayed lysis confers resistance to the nucleoside analogue 5-Fluorouracil and alleviates mutation accumulation in the single-stranded DNA bacteriophage x174. *J. Virol.* **88**, 5042-5049 (2014). |
| 51. | M. Pereira-Gómez, R. Sanjuán, Effect of mismatch repair on the mutation rate of bacteriophage ϕX174. *Virus Evolution* **1**, vev010 (2015). |
| 52. | C. Rajanna *et al.*, A strain of *Yersinia pestis* with a mutator phenotype from the Republic of Georgia. *FEMS Microbiol. Lett.* **343**, 113-120 (2013). |
| 53. | C. Riesenfeld, M. Everett, L. J. V. Piddock, B. G. Hall, Adaptive mutations produce resistance to ciprofloxacin. *Antimicrob. Agents Chemother.* **41**, 2059-2060 (1997). |
| 54. | C. W. Russell, M. A. Mulvey, The Extraintestinal Pathogenic *Escherichia coli* Factor RqlI Constrains the Genotoxic Effects of the RecQ-Like Helicase RqlH. *PLoS Pathogens* **11**, 1-29 (2015). |
| 55. | S. J. Schrag, P. A. Rota, W. J. Bellini, Spontaneous mutation rate of measles virus: direct estimation based on mutations conferring monoclonal antibody resistance. *J. Virol.* **73**, 51-54 (1999). |
| 56. | S. Shewaramani, T. J. Finn, S. C. Leahy, R. Kassen, P. B. Rainey, C. D. Moon, Anaerobically Grown *Escherichia coli* Has an Enhanced Mutation Rate and Distinct Mutational Spectra. *PLoS Genet.* 13(1): e1006570 (2017). |
| 57. | P. Siminoff, Development of Bacterial Resistance To Antibiotics. *J. Bacteriol.* **77**, 79-85 (1959). |
| 58. | M. Sussman, S. G. Bradley, Mutant yeast strains resistant to arsenate and azide. *J. Bacteriol.* **66**, 52-59 (1953). |
| 59. | C. Torres-Barceló, M. Kojadinovic, R. Moxon, R. C. MacLean, The SOS response increases bacterial fitness, but not evolvability, under a sublethal dose of antibiotic. *Proc. R. Soc. London, B* **282**, 20150885 (2015). |
| 60. | J. E. Turse, J. Pei, T. A. Ficht, Lipopolysaccharide-deficient *Brucella* variants arise spontaneously during infection. *Front. Microbiol.* **2**, 1-12 (2011). |
| 61. | A. J. Vogler *et al.*, Molecular Analysis of Rifampin Resistance in *Bacillus anthracis* and *Bacillus cereus*. *Antimicrob. Agents Chemother.* **46**, 511-513 (2002). |
| 62. | G. Wang *et al.*, Spontaneous Mutations That Confer Antibiotic Resistance in *Helicobacter pylori* Spontaneous Mutations That Confer Antibiotic Resistance in *Helicobacter pylori*. *Antimicrob. Agents Chemother.* **45**, 727-733 (2001). |
| 63. | T. Watanabe, T. Fukasawa, D. Ushiba, Probable absence of direct induction of bacterial resistance to streptomycin. *J. Bacteriol.* **73**, 770-777 (1957). |
| 64. | M. E. Watson, J. L. Burns, A. L. Smith, Hypermutable *Haemophilus influenzae* with mutations in mutS are found in cystic fibrosis sputum. *Microbiology* **150**, 2947-2958 (2004). |
| 65. | J. Werngren, S. E. Hoffner, Drug-susceptible *Mycobacterium tuberculosis* Beijing genotype does not develop mutation-conferred resistance to rifampin at an elevated rate. *J. Clin. Microbiol.* **41**, 1520-1524 (2003). |
| 66. | S. Wielgoss *et al.*, Mutation rate dynamics in a bacterial population reflect tension between adaptation and genetic load. *Proc. Natl. Acad. Sci. U. S. A.* **110**, 222-227 (2013). |
| 67. | C. Zeyl, M. Mizesko, J. A. G. M. De Visser, Mutational meltdown in laboratory yeast populations. *Evolution* **55**, 909-917 (2001). |
