# Supplementary material for: Spontaneous mutation rate is a plastic trait associated with population density across domains of life
Source: PLoS Biol. 2017 Aug 24;15(8):e2002731. doi: 10.1371/journal.pbio.2002731 (PMC5570273; doi:10.1371/journal.pbio.2002731)
Supplement: S2 Table — (DOCX) [file pbio.2002731.s013.docx]

S2 Table**.** **Bacterial and yeast strains.**

| **Strain** | **Genotype** | **Source or reference** |
| --- | --- | --- |
| *E. coli* MG1655 |  | Karina B. Xavier |
| *E. coli* JW3350-2 | F-, Δ*(araD-araB)567*, *ΔlacZ4787*(::rrnB-3*)*, λ-, Δ*dam-722*::kan, *rph-1*, Δ*(rhaD-rhaB)568*, *hsdR514* | Keio collection* |
| *E. coli* JW0221-1 | F-, Δ*(araD-araB)567*, *ΔlacZ4787*(::rrnB-3*)*, λ-, Δ*dinB749*::kan, *rph-1*, Δ*(rhaD-rhaB)568*, *hsdR514* | Keio collection* |
| *E. coli* JW2799-1 | F-, Δ*(araD-araB)567*, *ΔlacZ4787*(::rrnB-3*)*, λ-, Δ*mutH756*::kan, *rph-1*, Δ*(rhaD-rhaB)568*, *hsdR514* | Keio collection* |
| *E. coli* JW4128-1 | F-, Δ*(araD-araB)567*, *ΔlacZ4787*(::rrnB-3*)*, λ-, Δ*mutL720*::kan, *rph-1*, Δ*(rhaD-rhaB)568*, *hsdR514* | Keio collection* |
| *E. coli* JW3610-2 | F-, Δ*(araD-araB)567*, *ΔlacZ4787*(::rrnB-3*)*, λ-, Δ*mutM744*::kan, *rph-1*, Δ*(rhaD-rhaB)568*, *hsdR514* | Keio collection* |
| *E. coli* JW2703-2 | F-, Δ*(araD-araB)567*, *ΔlacZ4787*(::rrnB-3*)*, λ-, Δ*mutS738*::kan, *rph-1*, Δ*(rhaD-rhaB)568*, *hsdR514* | Keio collection* |
| *E. coli* JW0097-1 | F-, Δ*(araD-araB)567*, *ΔlacZ4787*(::rrnB-3*)*, λ-, Δ*mutT790*::kan, *rph-1*, Δ*(rhaD-rhaB)568*, *hsdR514* | Keio collection* |
| *E. coli* JW0097-3 | F-, Δ*(araD-araB)567*, *ΔlacZ4787*(::rrnB-3*)*, λ-, Δ*mutT790*::kan, *rph-1*, Δ*(rhaD-rhaB)568*, *hsdR514* | Keio collection* |
| *E. coli* JW2928-1 | F-, Δ*(araD-araB)567*, *ΔlacZ4787*(::rrnB-3*)*, λ-, Δ*mutY736*::kan, *rph-1*, Δ*(rhaD-rhaB)568*, *hsdR514* | Keio collection* |
| *E. coli* JW0704-1 | F-, Δ*(araD-araB)567*, *ΔlacZ4787*(::rrnB-3*)*, λ-, Δ*nei764*::kan, *rph-1*, Δ*(rhaD-rhaB)568*, *hsdR514* | Keio collection* |
| *E. coli* JW0194-1 | F-, Δ*(araD-araB)567*, *ΔlacZ4787*(::rrnB-3*)*, λ-, Δ*metI723*::kan, *rph-1*, Δ*(rhaD-rhaB)568*, *hsdR514* | Keio collection* |
| *P. aeruginosa* PAO1 |  | Johanna M. Schwingel |
| *S. cerevisiae* BY4742 |  | Daniela Delneri |
| *S. cerevisiae* Sigma1287b |  | Daniela Delneri |
| *S. cerevisiae* S288C |  | Daniela Delneri |
| *S. cerevisiae* Sigma1287b | *PCD1-*Δ | Daniela Delneri |
| *S. cerevisiae* BY4742 | *PCD1-*Δ | Daniela Delneri |
| *S. cerevisiae* Sigma1287b | *MLH1-*Δ | Daniela Delneri |

*Baba T, Ara T, Hasegawa M, Takai Y, Okumura Y, Baba M, et al. Construction of *Escherichia coli* K‐12 in‐frame, single‐gene knockout mutants: the Keio collection. Mol Syst Biol. 2006;2.
