# Supplementary material for: Spontaneous mutation rate is a plastic trait associated with population density across domains of life
Source: PLoS Biol. 2017 Aug 24;15(8):e2002731. doi: 10.1371/journal.pbio.2002731 (PMC5570273; doi:10.1371/journal.pbio.2002731)
Supplement: S3 Table — See Materials and Methods for more details about the analysis. Differences from the reference common to all Keio strains are not shown. Sequence data available at the European Nucleotide Archive (accession number ERP024110, http://www.ebi.ac.uk/ena/data/view/ERP024110). (DOCX) [file pbio.2002731.s014.docx]

S3 Table**. Breseq analysis of mutations identified in genome sequence for two Δ*mutT* Keio strains.**

See Materials and Methods for more details about the analysis. Differences from the reference common to all Keio strains are not shown. Sequence data available at the European Nucleotide Archive (accession number ERP024110, <http://www.ebi.ac.uk/ena/data/view/ERP024110>).

| Position | Mutation | Δ*mutT* 3795 | Δ*mutT 3796* | | annotation | | gene | | description |  |
| --- | --- | --- | --- | --- | --- | --- | --- | --- | --- | --- |
| 308335 | T>G |  | yes | E333A (GAG>GCG) | | NP_414825.1 < | | ECP production outer membrane protein | | |
| 1262200 | A>C |  | yes | L226L (CTT>CTG) | | NP_415726.1 < | | 4‑diphosphocytidyl‑2‑C‑methylerythritol kinase | | |
| 1344259 | A>C | Yes |  | S30A (TCT>GCT) | | NP_415800.1 < | | global regulator of transcription; DeoR family | | |
| 1513869 | T>G |  | yes | G13G (GGT>GGG) | | NP_415959.1 > | | putative ABC transporter permease | | |
| 1871819 | A>C | Yes |  | K479Q (AAA>CAA) | | NP_416299.1 > | | putative membrane‑anchored diguanylate cyclase | | |
| 2193558 | A>C | Yes |  | L204* (TTA>TGA) | | NP_416616.4 < | | antiporter inner membrane protein | | |
| 2405801 | A>C | Yes |  | Y281D (TAT>GAT) | | NP_416792.1 < | | transcriptional repressor of flagellar, motility and chemotaxis genes | | |
| 2406280 | A>C |  | yes | V121G (GTG>GGG) | | NP_416792.1 < | | transcriptional repressor of flagellar, motility and chemotaxis genes | | |
| 2959804 | A>C | Yes |  | L875L (CTT>CTG) | | NP_417299.1 < | | exonuclease V (RecBCD complex), gamma chain | | |
| 2994210 | T>G | Yes |  | I91R (ATA>AGA) | | pbl > | | pseudogene, peptidoglycan‑binding enzyme family | | |
| 3256922 | A>C |  | yes | N363K (AAT>AAG) | | YP_026203.3 < | | putative transporter | | |
| 3283396 | A>C |  | yes | E85A (GAA>GCA) | | NP_417606.1 > | | tagatose 6‑phosphate aldolase 1, kbaY subunit | | |
| 3441340 | T>G | Yes |  | A112A (GCA>GCC) | | NP_417755.1 < | | 30S ribosomal subunit protein S4 | | |
| 3828787 | A>C | Yes |  | intergenic (‑122/‑158) | | NP_418110.1 < / > NP_418111.1 | | glutamate transporter/xanthine permease | | |
| 3859103 | A>C |  | yes | F338L (TTT>TTG) | | NP_418135.1 < | | putative transporter | | |
| 3992438 | T>G | Yes |  | L429* (TTA>TGA) | | NP_418250.1 > | | adenylate cyclase | | |
| 3995699 | A>C |  | yes | E39D (GAA>GAC) | | NP_418255.1 > | | DUF484 family protein | | |
| 4009304 | T>G |  | yes | F45L (TTT>TTG) | | YP_026266.1 > | | lysophospholipase L2 | | |
| 4087643 | T>G |  | yes | I214M (ATT>ATG) | | NP_418332.1 > | | DUF3829 family lipoprotein | | |
| 4187714 | A>C |  | yes | K789Q (AAA>CAA) | | NP_418415.1 > | | RNA polymerase, beta prime subunit | | |
| 4361043 | T>G |  | yes | N298H (AAT>CAT) | | NP_418557.1 < | | cadBA operon transcriptional activator | | |
| 4463045 | A>C |  | yes | intergenic (‑385/+9) | | NP_418659.1 < / < NP_418660.1 | | anaerobic ribonucleoside‑triphosphate reductase/trehalose‑6‑P hydrolase | | |
