# Supplementary material for: Spontaneous mutation rate is a plastic trait associated with population density across domains of life
Source: PLoS Biol. 2017 Aug 24;15(8):e2002731. doi: 10.1371/journal.pbio.2002731 (PMC5570273; doi:10.1371/journal.pbio.2002731)
Supplement: S4 Table — (DOCX) [file pbio.2002731.s015.docx]

S4 Table**. Detailed description of the columns in the raw data file S1_Data.csv**

| “ID" | A unique identifier for individual data points. |
| --- | --- |
| "figure" | The figure in which the row of data is used. |
| "mutation_rate" | The estimated mutation rate per genome per generation multiplied by 10^9^. |
| "estimator" | Name of mutation rate estimation method used for estimating “m” |
| "domain" | The domain that organism, used in the fluctuation test, belongs to. |
| "organism" | The organism used in the fluctuation test. |
| "strain" | The strain used (BY4742, Keio, MG1655, REL606, REL607, PAO1, S288C and Sigma_1278b corresponding to strains *S. cerevisiae* BY4742, strains from Keio collection, *E. coli* MG1655, *E. coli* B REL606, *E. coli* B REL607, *P. aeruginosa* PAO1, *S. cerevisiae* S288C and *S. cerevisiae* Sigma_1278b, respectively). See S2 Table for more details. |
| "genotype" | The genotype of the strain (BY4742, S288C, Sigma_1278b, MLH1_sigma, PCD1_sigma, PCD1_by, MG1655, PAO1, Rif+_Ara-, Rif+_Ara+, dam, dinB, metI, mutH, mutL, mutM, mutS, mutT1, mutT2, nei and mutY, corresponding to *S. cerevisiae* BY4742, S288C, Sigma_1278b, Sigma_1278b *MLH1-*Δ, Sigma_1278b *PCD1-*Δ, BY4742 *PCD1-*Δ, *E. coli* MG1655, *P. aeruginosa* PAO1, *E. coli* B REL606, *E. coli* B REL607, and gene knockouts from Keio collection. See S2 Table and main text for more details. |
| "recalculation" | This column shows if mutation rate used in Fig 1 was recalculated. |
| "Authors" | List of authors of the paper containing mutation rates used in Fig 1. |
| "Title" | Title of the paper containing mutation rates used in Fig 1. |
| "Year" | Publication year of the paper containing mutation rates used in Fig 1. |
| "Journal" | Journal where the paper containing mutation rates used in Fig 1 was published. |
| "environment" | Medium used for growing parallel cultures. |
| "marker" | Phenotypic (selective) marker used in the fluctuation test. |
| "researcher" | The person who actually conducted the fluctuation test (RK, HR, CH and KF correspond to Rok Krašovec, Huw Richards, Charlie Hatcher and Katy J. Faulkner). |
| "block" | The number of the experimental block in which the fluctuation test was done. |
| "plate_ID" | A unique identifier of an individual 96-deep-well plate used in the fluctuation test. |
| "included" | when “m” is >30 or <0.3 is designated as “excluded” otherwise “included”. |
| "culture_volume" | The initial volume of parallel cultures. |
| "glucose_mg_per_L" | The initial concentration of glucose in “environment”. |
| "percentage_of_YP" | The proportion of yeast extract/peptone in “environment”. |
| "N0" | The initial population size of cells in parallel cultures. |
| "Nt" | The population size at the end of the culture period estimated via colony forming units averaged over three parallel cultures. |
| "SD_Nt" | The standard deviation of “Nt”. |
| "D" | The estimated number of cells per ml at the end of the culture period calculated with colony forming units and averaged over three parallel cultures. |
| "LUM" | Net luminescence (LUM_510_-LUM_0.5_) in arbitrary units measured with luminometer using Bac-Titer Glo kit. |
| "CC" | Cell counts measured with haemocytometer. |
| "LUM_gross" | Gross luminescence (LUM_510_) in arbitrary units measured with luminometer using Bac-Titer Glo kit. |
| "generations" | The number of generations at the end of the incubation of parallel cultures calculated as (log(“Nt”/”N0”))/log2 |
| “generation_time” | “generations” divided by “incubation_time”. |
| "Ne" | Effective population size |
| "m" | Number of mutational events calculated with Ma-Sandri-Sarkar estimator. |
| "SD_m" | Standard deviation of the number of mutational events calculated with the equation 27* using “m” and “C_cultures*”*. |
| "C_cultures" | The number of parallel cultures used for estimating “m”. |
| ”upper_bound” | Upper bound of the mutation rate is calculated as the upper bound of “m” (95%, CI) divided by ”Nt”. |
| “lower_bound” | Lower bound of the mutation rate is calculated as the lower bound of “m” (95%, CI) divided by ”Nt”. |
| “W” | Relative fitness |
| “host” | Names of virus host cell lines from Sanjuan *et al*. 2010** |
| "culture_1" | Number of observed mutants in a parallel culture no. 1. |
| "culture_2" | Number of observed mutants in a parallel culture no. 2. |
| "culture._3" | Number of observed mutants in a parallel culture no. 3. |
| "culture_4" | Number of observed mutants in a parallel culture no. 4. |
| "culture_5" | Number of observed mutants in a parallel culture no. 5. |
| "culture_6" | Number of observed mutants in a parallel culture no. 6. |
| "culture_7" | Number of observed mutants in a parallel culture no. 7. |
| "culture_8" | Number of observed mutants in a parallel culture no. 8. |
| "culture_9" | Number of observed mutants in a parallel culture no. 9. |
| "culture_10" | Number of observed mutants in a parallel culture no. 10. |
| "culture_11" | Number of observed mutants in a parallel culture no. 11. |
| "culture_12" | Number of observed mutants in a parallel culture no. 12. |
| "culture_13" | Number of observed mutants in a parallel culture no. 13. |
| "culture_14" | Number of observed mutants in a parallel culture no. 14. |
| "culture_15" | Number of observed mutants in a parallel culture no. 15. |
| "culture_16" | Number of observed mutants in a parallel culture no. 16. |
| "culture_17" | Number of observed mutants in a parallel culture no. 17. |

*Foster PL. Methods for determining spontaneous mutation rates. Methods Enzymol. 2006;409:195-213.

**Sanjuan R, Nebot MR, Chirico N, Mansky LM, Belshaw R. Viral mutation rates. J Virol. 2010;84:9733-48.
