# Supplementary material for: Spontaneous mutation rate is a plastic trait associated with population density across domains of life
Source: PLoS Biol. 2017 Aug 24;15(8):e2002731. doi: 10.1371/journal.pbio.2002731 (PMC5570273; doi:10.1371/journal.pbio.2002731)
Supplement: S1 Text — Descriptions of all statistical models, including ANOVA tables and diagnostic plots. (DOCX) [file pbio.2002731.s016.docx]

# Supplementary Text – Statistical Models

## Model S-I

The model fitted to data presented in Fig 1 (analysis of published mutation rates) is the log_2_ transformed mutation rates from the published literature as a function of the mean-centred log_2_ transformed final population density, *D* (fixed effect). The effect of the organism (26 levels), culture environment (44 levels), the marker and its concentration (70 levels) and the article the estimate was published in (68 levels) had on the intercept were all initially included as, partially crossed, random effects. We also initially included random slopes effects for all these factors. Because of known differences in estimator accuracy we also allowed for the possibility of different levels of variance for the different estimators used to calculate mutation rate. To do this we created indicator variables for the four different estimators used for calculating mutation rate. We then incorporated each individual indicator variable within the model allowing a different individual level random effect for each estimation method. The spaMM package allows for incorporating a correlation matrix. We used the correlation expected under a Brownian model of evolution across the phylogenetic tree described in the Materials and Methods (S1 Fig), calculated via the ape package*, using *V_ij_* = *γ* x *t_a_*, where *t_a_* is the distance between the root and last common ancestor for taxa *i* and *j* and *γ* is a constant. We used Akaike Information Criterion (AIC) to reduce this maximal model by removing unnecessary effects. During model simplification, models with each of the above random effects on the intercept and either organism or published article on slope were equivalent (difference of 0.3 in AIC between models). The final minimal adequate model reported consists of random effects of culture environment, organism, marker and its concentration and published article on the intercept, and a random effect of organism on the slope with *D*. Further details are given in ANOVA table below. The proportion of variance explained by the fixed effect (*D*) was calculated based on the equations given in Nakagawa and Schielzeth (2013)**. Specifically, we calculated the variance explained by the fixed effect (***σ*^2^***_f_* = 8.05), the variance explained by the random effects (the sum of the variance explained by each random effect *l*, ***σ*^2^***_L_* = 12.02) and the total variance (the sum of the variance explained by the fixed effect, random effects and the residual variance i.e. *σ*^2^*_t_* = *σ*^2^*_f_* + *σ*^2^*_L_* + *σ*^2^_ε_ = 20.68) to give the proportion of variance explained by the fixed effect of *D*, accounting for the random effects: ***σ*^2^***_f_* / (***σ*^2^***_t_* - ***σ*^2^***_L_*) = 0.929 Explicit inclusion of genome size (median total length in Mb as listed for each species name at www.ncbi.nlm.nih.gov/genome on 2^nd^ June 2017) as a fixed effect in this model has little effect (difference of 0.03 in AIC between models; proportion of variance explained by *D* and genome size together, having accounted for variance explained by the random effects, = 0.930).

*Paradis E, Claude J, Strimmer K. APE: Analyses of phylogenetics and evolution in R language. Bioinformatics. 2004;20:289-90.

**Nakagawa S, Schielzeth H. A general and simple method for obtaining R2 from generalized linear mixed-effects models. Methods Ecol Evol. 2013;4:133-42.

**ANOVA table and fitted values for** Model S-I (Fig 1).

See Materials and Methods for more details.

|  | Value | SE | |
| --- | --- | --- | --- |
| *Fixed Effects*  Intercept | 5.1 | | 0.52 |
| log_2_(*D*)*_centred_*  *Random Effects*  Environment_Intercept_  Marker & Concentration_Intercept_  Paper_Intercept_  Organism_Intercept_  Organism_slope_  Maximum Likelihood estimator  *p0* estimator  Mean estimator  Residual (median estimator) | -0.67 | | 0.085  SD  0.75  4.8  2.4  1.1  0.04  4.3x10^-9^  0.23  2.8  0.61 |

## Model S-II

The model shown in Fig 2A (wild-type bacterial strains) fits log_2_ mutation rate against mean-centred log_2_ *D* (estimated via luminescence, see Model S-IV below) allowing for differences in intercept and slope among the three treatments (different genotype/marker combinations). For this and all subsequent models, an initial model was fit by restricted maximum likelihood (REML) including all fixed effects (in this case *D*, treatment and their interaction) and all random effects (experimental plate nested within experimental block nested within experimenter, each affecting the intercept). A series of variants of this model was constructed allowing differences in variance (i.e. heteroscedasticity) associated with one or two covariates.

Potential variance covariates considered were: experimental block, organism, strain and genotype identity, selective marker and liquid growth media used, all treated as discrete effects with a different variance at each level. Continuous variance covariates allowed variance to change as a power function of the covariate. Potential continuous variance covariates considered were: the fitted values of the response variable, the initial population size (*N_0_*), the number of mutational events estimated (*m*), the coefficient of variation (CV) and standard deviation in that estimate, the initial glucose concentration, the final population size (*N_t_*) and its standard deviation, *D* (estimated by colony forming units, CFU, cell count, CC, or net luminescence, LUM, as available), net luminescence per cell (LUM/*N_t_*), gross luminescence, absolute fitness, the number of generations, the generation time, the percentage of YP, phi (1-N_t_/N_0_), number, volume and incubation time of parallel cultures used in the fluctuation test, proportion of weight remaining following evaporation during the growth of parallel cultures in the liquid media, upper bound, lower bound and range of the mutation rate estimate.

The model variant with the lowest AIC was then chosen. In this case this model allowed variance to change with [standard deviation of the estimated number of mutational events x number of parallel cultures]^1.3^. The two slopes of mutation rate with *D* estimated for *Escherichia coli* at different genotypic markers were very similar and this model was therefore simplified by combining these slopes to estimate a single value. No further simplification of the fixed effects was possible without significantly reducing the goodness of fit of the model. Here and below, a significant reduction in the goodness of fit was taken to mean *P* < 0.05 by likelihood ratio test, comparing models fit with and without particular fixed effects by maximum likelihood. Further details are given in ANOVA table and in diagnostic plots.

**ANOVA table and fitted values for** Model S-II **(Fig 2A).**

See Materials and Methods for more details.

|  | Degrees of freedom | Value | SE | *F* | *P* |
| --- | --- | --- | --- | --- | --- |
| Intercept (MG1655_rifampicin50) | 1 | 3.3 | 0.21 | 135 | 2.8×10^-26^ |
| log_2_(D)*_centred_* | 1 | -0.68 | 0.059 | 129 | 1.7×10^-18^ |
| genotype_marker (MG1655_nalidixic_acid30) | 2 | -4.1 | 0.32 | 88 | 1.8×10^-9^ |
| (PAO1_rifampicin50) |  | -1.9 | 0.32 |  |  |
| log_2_(D)*_centred_*:genotype_marker | 1 | 0.53 | 0.14 | 15 | 2.7×10^-4^ |


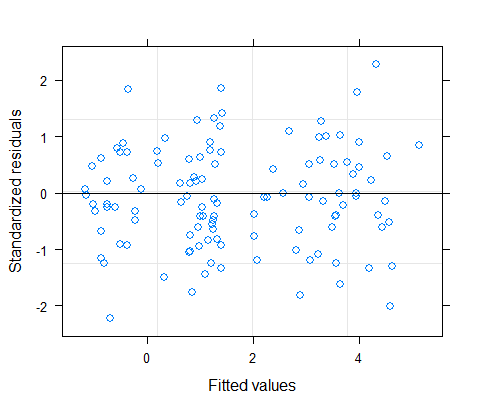

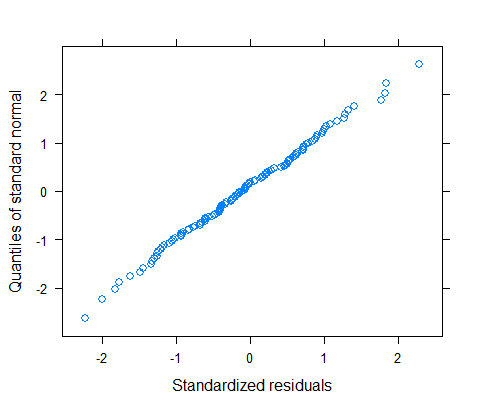


**Diagnostic plots for** Model S-II.

Standardised residuals by fitted values and normal quantile-quantile plot of standardised residuals.

## Model S-III

The model shown in Fig 2B (wild-type yeast strains) fits log_2_ mutation rate against mean-centred log_2_ *D* (estimated from direct cell counts), interacting with fixed effects of genotype treatments. Random effects of experimental plate nested within experimental block on the intercept were also included. The best model allowed variance to change with both the [culture volume]^2.7^ and the experimental block. The two slopes of mutation rate with *D* estimated for strains S288c and Sigma1278b were very similar, and this model was therefore simplified as above to combine these slopes to estimate a single value. No further simplification of the fixed effects was possible without significantly reducing the goodness of fit of the model. Further details are given in ANOVA table and in diagnostic plots.

**ANOVA table and fitted values for** Model S-III **(Fig 2B).**

See Materials and Methods for more details.

|  | Degrees of freedom | Value | SE | *F* | *P* |
| --- | --- | --- | --- | --- | --- |
| Intercept (BY 4742) | 1 | 10 | 0.49 | 309 | 6.0×10^-35^ |
| log_2_(D)*_centred_* | 1 | -0.32 | 0.038 | 313 | 5.5×10^-13^ |
| genotype (S288C) | 2 | -5.8 | 1.1 | 690 | 5.3×10^-4^ |
| (Sigma_1278b) |  | -4.7 | 0.13 |  |  |
| log_2_(D)*_centred_*:genotype | 1 | -0.66 | 0.13 | 25 | 3.6×10^-6^ |


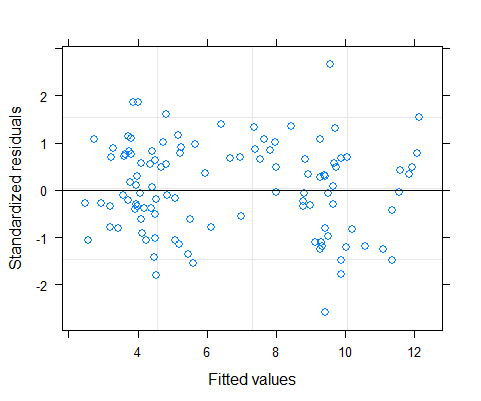

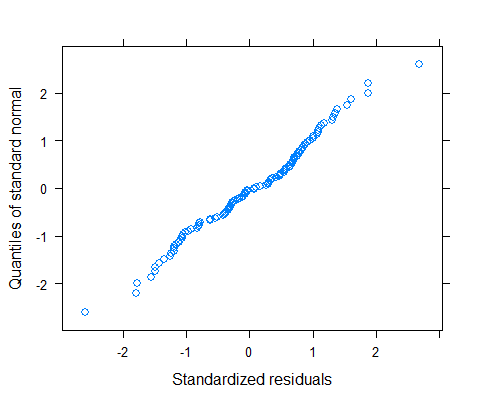


**Diagnostic plots for** Model S-III.

Standardised residuals by fitted values and normal quantile-quantile plot of standardised residuals.

## Model S-IV

The model shown in S3 Fig (calibration curve) fits log_2_ *D* (measured using colony forming units) against mean-centred log_2_ of luminescence (in arbitrary units from the BacTiter-Glo assay, LUM), organism (i.e. species) and their interaction and random effects of genotype on the slope and intercept and of experimental plate nested within experimental block on the intercept. The best model allowed variance to change with [experimental block] x [fitted values of *D*]^-1.4^. No simplification of the fixed effects was possible without significantly reducing the goodness of fit of the model. This model was used to calibrate the luminescence values to give the population densities used in Model S-II, Model S-VII, Model S-VIII and Model S-X (allowing a different calibration curve for each genotype). Further details are given in ANOVA table and in diagnostic plots.

**ANOVA table and fitted values for** Model S-IV **(**S3 Fig**).**

See Materials and Methods for more details.

|  | Degrees of freedom | Value | SE | *F* | *P* |
| --- | --- | --- | --- | --- | --- |
| Intercept (*E. coli*) | 1 | 27 | 0.049 | 349696 |  |
| log_2_(LUM)*_centred_* | 1 | 0.70 | 0.049 | 243 | 1.4×10^-33^ |
| organism(*P. aeruginosa*) | 1 | 0.48 | 0.17 | 5.6 | 0.038 |
| log_2_(LUM)*_centred_*:organism | 1 | 0.38 | 0.17 | 5.0 | 0.027 |


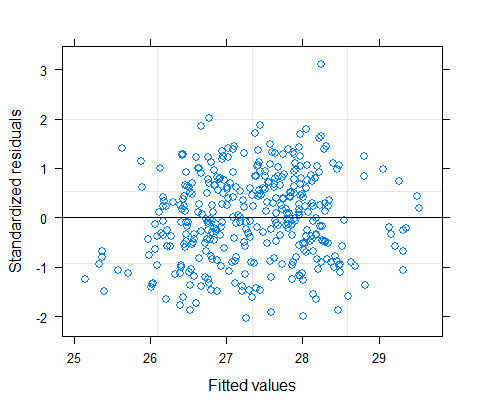

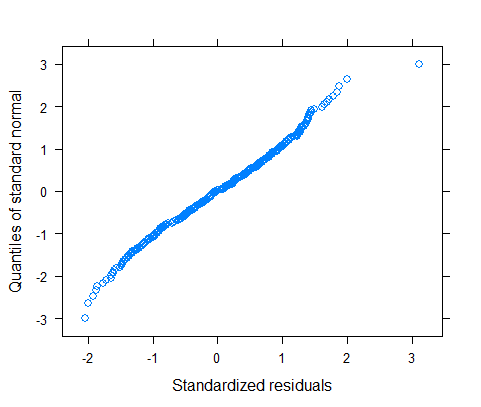


**Diagnostic plots for** Model S-IV.

Standardised residuals by fitted values and normal quantile-quantile plot of standardised residuals.

## Model S-V

The model shown in S4A Fig (wild-type bacterial strains) is very similar to Model S-II**,** but using *D* estimated by CFU rather than LUM. The best model allowed variance to change with [estimated number of mutational events]^-0.38^ and [number of parallel cultures]^1.8^. The two slopes of mutation rate with *D* estimated for *Escherichia coli* at different genotypic markers were very similar and this model was therefore simplified by combining these slopes to estimate a single value. No further simplification of the fixed effects was possible without significantly reducing the goodness of fit of the model. Further details are given in ANOVA table and in diagnostic plots.

**ANOVA table and fitted values for** Model S-V (**S4**A **Fig**).

See Materials and Methods for more details.

|  | Degrees of freedom | Value | SE | *F* | *P* |
| --- | --- | --- | --- | --- | --- |
| Intercept (MG1655_rifampicin50) | 1 | 3.3 | 0.14 | 302 | 3.3×10^-37^ |
| log_2_(D)*_centred_* | 1 | -0.73 | 0.053 | 165 | 6.5×10^-23^ |
| genotype_marker (MG1655_nalidixic_acid30) | 2 | 4.2 | 0.25 | 150 | 3.1×10^-11^ |
| (PAO1_ rifampicin50) |  | 2.1 | 0.27 |  |  |
| log_2_(D)*_centred_*:genotype_marker | 1 | 0.63 | 0.13 | 25 | 3.4×10^-6^ |


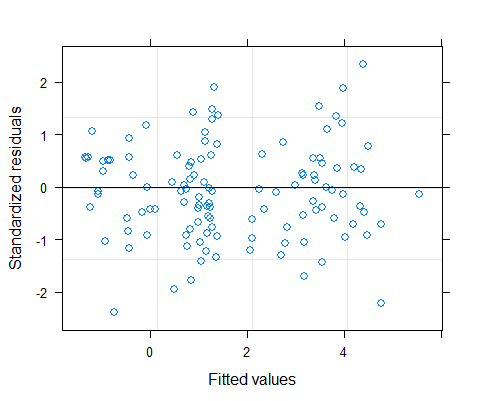

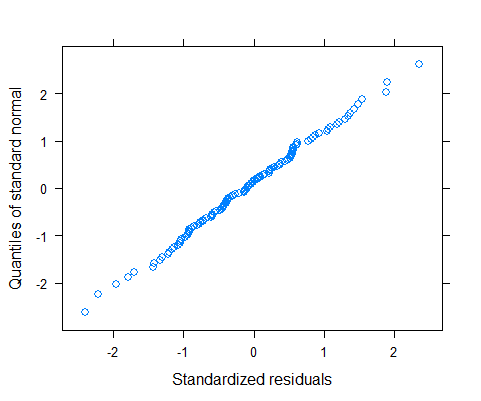


**Diagnostic plots for** Model S-V.

Standardised residuals by fitted values and normal quantile-quantile plot of standardised residuals.

## Model S-VI

The model shown in S4B Fig (wild-type yeast strains) is very similar to Model S-III but using *D* estimated by CFU. The best model allowed variance to change with [generation time x culture volume]^0.66^. The two slopes of mutation rate with *D* estimated for strains BY4742 and Sigma1278b were very similar, and this model was therefore simplified as above to combine these slopes to estimate a single value. No further simplification of the fixed effects was possible without significantly reducing the goodness of fit of the model. Further details are given in ANOVA table and in diagnostic plots.

**ANOVA table and fitted values for** Model S-VI **(S4B Fig).**

See Materials and Methods for more details.

|  | Degrees of freedom | Value | SE | *F* | *P* |
| --- | --- | --- | --- | --- | --- |
| Intercept (S288C) | 1 | 5.3 | 0.77 | 349 | 3.9×10^-10^ |
| log_2_(D)*_centred_* | 1 | -0.69 | 0.16 | 112 | 3.2×10^-5^ |
| genotype (BY4742) | 2 | 4.6 | 0.89 | 225 | 1.4×10^-6^ |
| (Sigma_1278b) |  | 0.28 | 0.89 |  |  |
| log_2_(D)*_centred_*:genotype | 1 | 0.48 | 0.17 | 8.1 | 0.0052 |


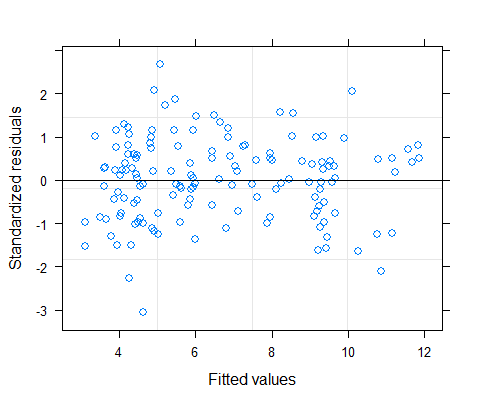

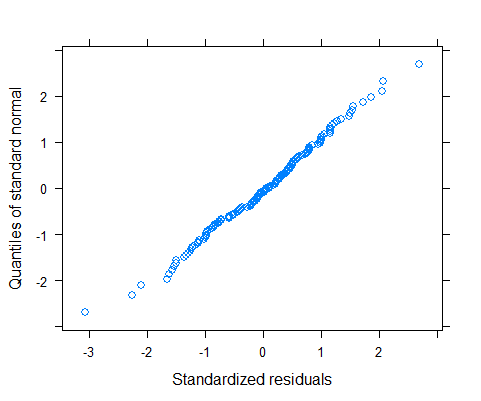


**Diagnostic plots for** Model S-VI.

Standardised residuals by fitted values and normal quantile-quantile plot of standardised residuals.

## Model S-VII

The model shown in Fig 3 (various bacterial gene knockouts) fits log_2_ mutation rate against mean-centred log_2_ population *D* (estimated via luminescence, see Model S-IV), interacting with a fixed effect of “MMR” (*mutH* / *mutL* / *mutS*) “*dinB”*, “*dam”, “nei” and “metI”*. The model includes random effects of experimental plate nested within experimental block, and allowing variance to change as [fitted values of the mutation rate]^-5.1^ x [upper bound of the mutation rate]^-1.7^. Slopes of mutation rate with *D* estimated for “*dinB*”, “*dam*” and “*metI*” were very similar and this model was therefore simplified by combining these slopes to estimate a single value. Further details are given in ANOVA table and in diagnostic plots.

**ANOVA table and fitted values for** Model S-VII (Fig 3).

See Materials and Methods for more details.

|  | Degrees of freedom | Value | SE | *F* | *P* |
| --- | --- | --- | --- | --- | --- |
| Intercept (Δ*dam*) | 1 | 4.2 | 0.12 | 8272 | 6.5×10^-55^ |
| log_2_(D)*_centred_* | 1 | -1.1 | 0.067 | 232 | 4.2×10^-30^ |
| system (Δ*dinB*) | 4 | -0.019 | 0.15 |  |  |
| (Δ*metI*) |  | -0.34 | 0.25 |  |  |
| (MMR) |  | 1.55 | 0.14 |  |  |
| (Δ*nei*) |  | -0.30 | 0.23 |  |  |
| log_2_(D)*_centred_*:(MMR) | 2 | 0.93 | 0.082 | 97 | 3.5×10^-19^ |
| log_2_(D)*_centred_*:(nei) |  | -0.40 | 0.14 |  |  |


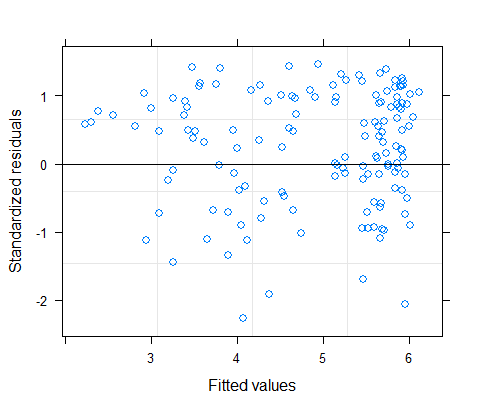

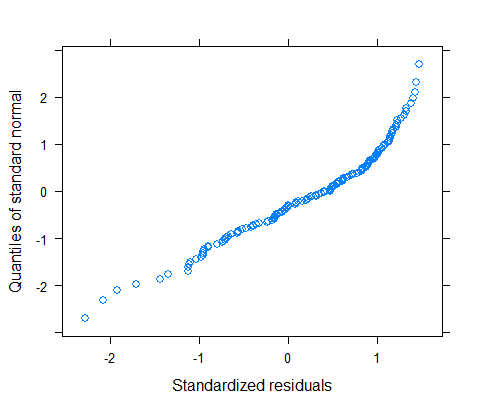


**Diagnostic plots for** Model S-VII.

Standardised residuals by fitted values and normal quantile-quantile plot of standardised residuals.

## Model S-VIII

The initial model fit to the data in Fig 4A (*E. coli* Δ*mutT* strains) was similar to Model S-II above with fixed effects of *D* (estimated with luminescence, see Model S-IV),genotype and their interaction and random effects on the intercept of plate nested within block. However, model simplification proceeded further to a minimal adequate model with no effect of *D* on mutation rate at all, the only significant fixed effect being a difference in intercept between the two strains considered. Variance changed as [fitted values of the mutation rate]^6.5^ x [upper bound of the mutation rate]^-2.2^. Further details are given in ANOVA table and in diagnostic plots.

**ANOVA table and fitted values for** Model S-VIII **(Fig 4A).**

See Materials and Methods for more details.

|  | Degrees of freedom | Value | SE | *F* | *P* |
| --- | --- | --- | --- | --- | --- |
| Intercept (Δ*mutT:*JW0097-1) | 1 | 5.4 | 0.17 | 929 | 4.4×10^-31^ |
| genotype (Δ*mutT:*JW0097-3) | 1 | -1.1 | 0.12 | 90 | 5.1×10^-12^ |


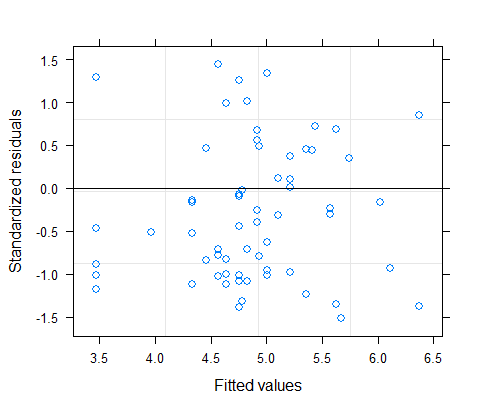

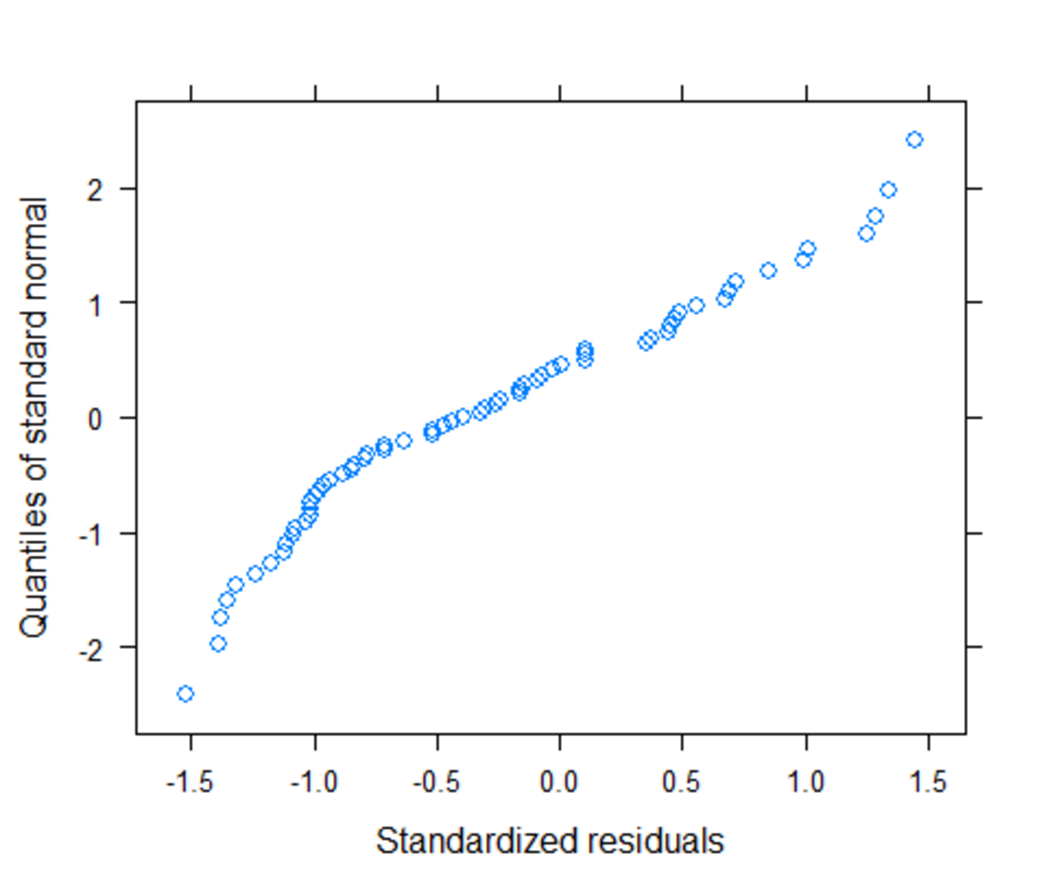


**Diagnostic plots for** Model S-VIII.

Standardised residuals by fitted values and normal quantile-quantile plot of standardised residuals.

## Model S-IX

The model shown in S8A Fig (*E. coli* Δ*mutT* strains) fits log_2_ mutation rate against mean-centred log_2_ *D* (estimated with CFU), genotype and their interaction and random effects on the intercept of plate nested within block. Model simplification proceeded to a minimal adequate model with no effect of *D* on mutation rate at all, the only significant fixed effect being a difference in intercept between the two strains considered. Variance changed with [fitted values of the mutation rate]^5.3^ and [lower bound of the mutation rate]^-1.5^. No further simplification of the fixed effects was possible without significantly reducing the goodness of fit of the model. Further details are given in ANOVA table and in diagnostic plots.

**ANOVA table and fitted values for** Model S-IX (S8A Fig).

See Materials and Methods for more details.

|  | Degrees of freedom | Value | SE | *F* | *P* |
| --- | --- | --- | --- | --- | --- |
| Intercept (Δ*mutT:*JW0097-1) | 1 | 5.5 | 0.16 | 1041 | 5.9×10^-32^ |
| genotype (Δ*mutT:*JW0097-3) | 1 | -1.1 | 0.12 | 87 | 8.6×10^-12^ |


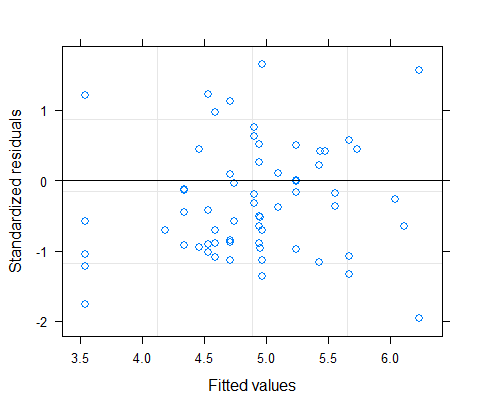

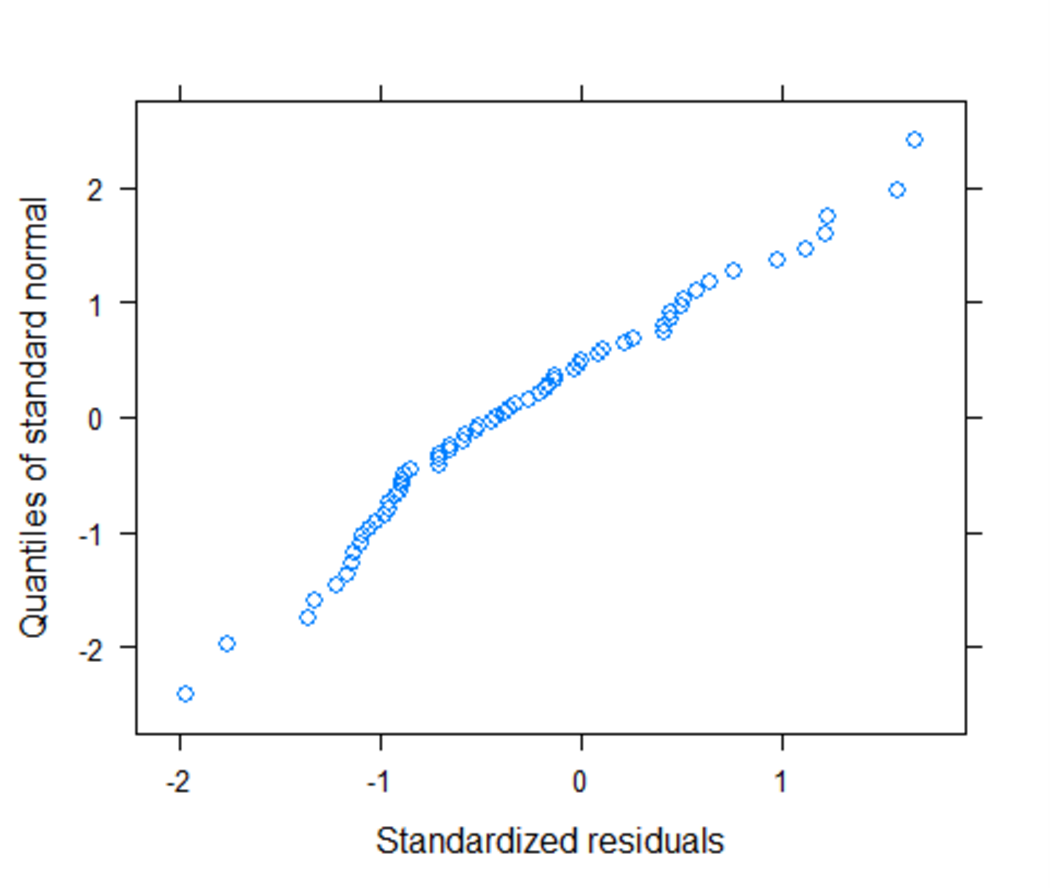


**Diagnostic plots for** Model S-IX.

Standardised residuals by fitted values and normal quantile-quantile plot of standardised residuals.

## Model S-X

The model fit to the data in Fig 4B (*E. coli* Δ*mutM* and Δ*mutY* strains) is similar to Model S-VIII above with fixed effects of *D* (estimated with luminescence, see Model S-IV), genotype and their interaction and random effects on the intercept of plate nested within block. The two slopes were very similar and this model was therefore simplified by combining these slopes to estimate a single value. Variance changed with [standard deviation of the estimated number of mutational events x genotype]. Further details are given in ANOVA table and in diagnostic plots.

**ANOVA table and fitted values for** Model S-X **(Fig 4B).**

See Materials and Methods for more details.

|  | Degrees of freedom | Value | SE | *F* | *P* |
| --- | --- | --- | --- | --- | --- |
| Intercept (Δ*mutM*) | 1 | 4.7 | 0.038 | 38081 | 2.7×10^-45^ |
| log_2_(D)*_centred_* | 1 | -1.1 | 0.041 | 716 | 2.3×10^-24^ |
| genotype (Δ*mutY*) | 1 | -0.40 | 0.050 | 62 | 4.8×10^-9^ |


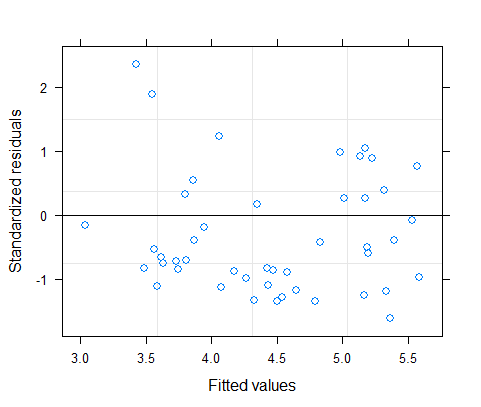

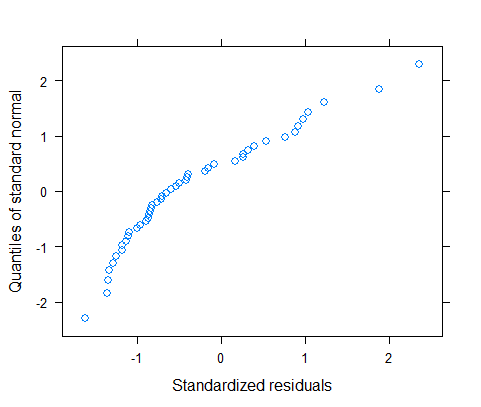


**Diagnostic plots for** Model S-X.

Standardised residuals by fitted values and normal quantile-quantile plot of standardised residuals.

## Model S-XI

The model shown in S8B Fig (*E. coli* Δ*mutM* and Δ*mutY* strains) fits log_2_ mutation rate against mean-centred log_2_ *D* (estimated with CFU), genotype and their interaction and random effects on the intercept of plate nested within block. Variance changed with [fitted values of the mutation rate]^-7.0^ and [upper bound of the mutation rate]^3.1^. No further simplification of the fixed effects was possible. Further details are given in ANOVA table and in diagnostic plots.

**ANOVA table and fitted values for** Model S-XI (S8B Fig).

See Materials and Methods for more details.

|  | Degrees of freedom | | Value | | SE | | *F* | | *P* | |
| --- | --- | --- | --- | --- | --- | --- | --- | --- | --- | --- |
| Intercept (Δ*mutM*) | | 1 | | 4.1 | | 0.13 | | 906 | | 1.8×10^-25^ |
| log_2_(D)*_centred_* | | 1 | | -0.97 | | 0.075 | | 248 | | 3.5×10^-14^ |
| genotype (Δ*mutY*)  log_2_(D)*_centred_*:genotype | | 1  1 | | -0.20  0.21 | | 0.070  0.11 | | 4.4  4.0 | | 0.0437  0.0528 |


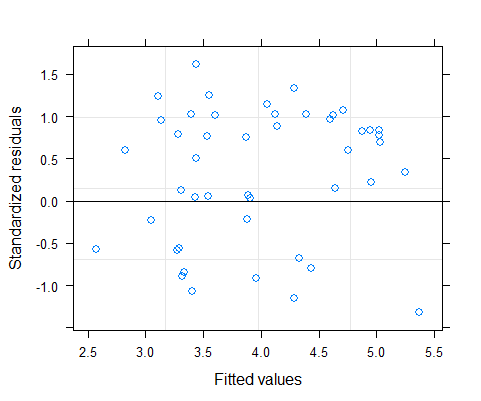

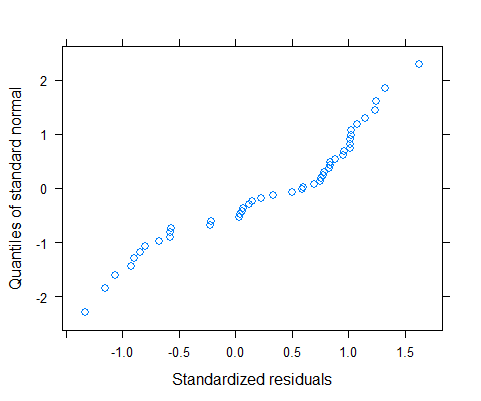


**Diagnostic plots for** Model S-XI.

Standardised residuals by fitted values and normal quantile-quantile plot of standardised residuals.

## Model S-XII

The initial model fit to the data in Fig 4C (*S. cerevisiae* *PCD1*-Δ strains) fits log_2_ mutation rate against mean-centred log_2_ *D* (estimated with CFU), genotype and their interaction and random effects on the intercept of plate nested within block). However, model simplification removed all effects of genotype and *D*. Variance changed as [fitted values of the mutation rate]^11^ and [upper bound of the mutation rate]^-1.7^. Further details are given in ANOVA table and in diagnostic plots.

**ANOVA table and fitted values for** Model S-XII **(Fig 4C) and** Model S-XIII (S8C Fig).

See Materials and Methods for more details.

|  | Degrees of freedom | Value | SE | *F* | *P* |
| --- | --- | --- | --- | --- | --- |
| Intercept | 1 | 12 | 0.72 | 270 | 3.1×10^-16^ |


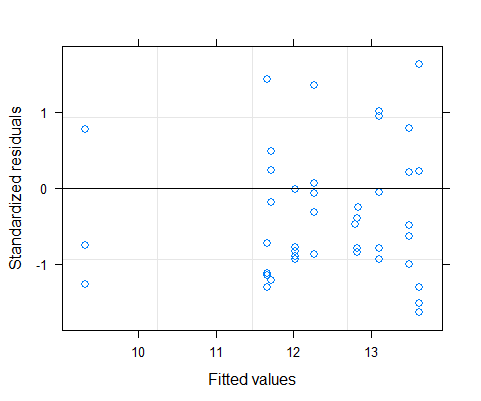

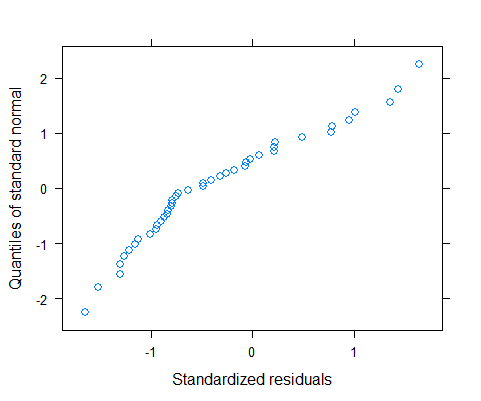


**Diagnostic plots for** Model S-XII **and** Model S-XIII.

Standardised residuals by fitted values and normal quantile-quantile plot of standardised residuals.

## Model S-XIII

The initial model fit to the data in S8C Fig (*S. cerevisiae* *PCD1*-Δ strains) fit log_2_ mutation rate against mean-centred log_2_ *D* (estimated from direct cell counts), genotype and their interaction and random effects on the intercept of plate nested within block. However, this simplified to a model identical with Model S-XII. ANOVA table and diagnostic plots are the same as for the Model S-XII.

## Model S-XIV

The model shown in Fig 4D (*S. cerevisiae* *MLH1*-Δ strain) fits log_2_ mutation rate against mean-centred log_2_ *D* (estimated with CFU) with random effects on the intercept of plate nested within block. Variance changed as [fitted values of the mutation rate]^4.0^ and [lower bound of the mutation rate]^-1.7^. No further simplification of the fixed effects was possible without significantly reducing the goodness of fit of the model. Further details are given in ANOVA table and in diagnostic plots.

**ANOVA table and fitted values for** Model S-XIV **(Fig 4D).**

See Materials and Methods for more details.

|  | Degrees of freedom | Value | SE | *F* | *P* |
| --- | --- | --- | --- | --- | --- |
| Intercept | 1 | 3.6 | 0.82 | 18 | 3.1×10^-4^ |
| log_2_(D)*_centred_* | 1 | -1.2 | 0.10 | 146 | 2.2×10^-10^ |


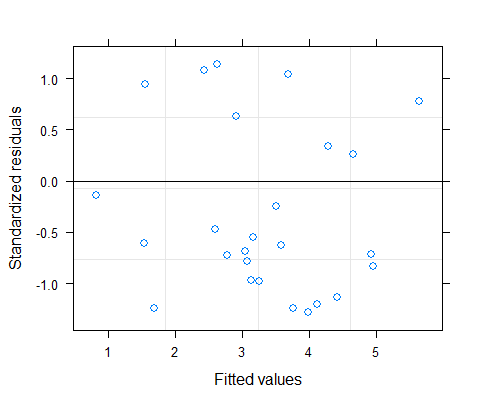

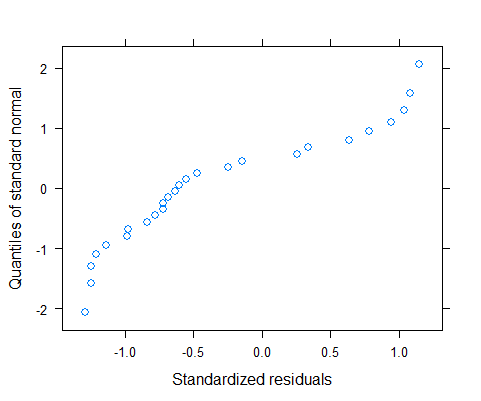


**Diagnostic plots for** Model S-XIV.

Standardised residuals by fitted values and normal quantile-quantile plot of standardised residuals.

## Model S-XV

The model shown in S8D Fig (*S. cerevisiae* *MLH1*-Δ strain) fits log_2_ mutation rate against mean-centred log_2_ *D* (estimated from direct cell counts) with random effects on the intercept of plate nested within block. Variance changed as [estimated standard deviation of *N_t_* x fitted values of the mutation rate]^-0.73^. No simplification of the fixed effects was possible without significantly reducing the goodness of fit of the model. Further details are given in ANOVA table and in diagnostic plots.

**ANOVA table and fitted values for** Model S-XV (S8D Fig**).**

See Materials and Methods for more details.

|  | Degrees of freedom | Value | SE | *F* | *P* |
| --- | --- | --- | --- | --- | --- |
| Intercept | 1 | 3.0 | 3.3 | 0 | 0.99 |
| log_2_(D)*_centred_* | 1 | -0.80 | 0.23 | 12 | 0.0060 |


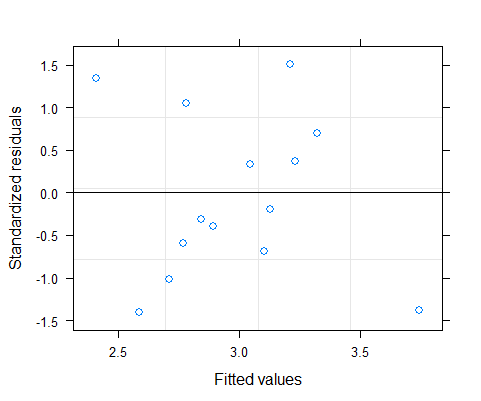

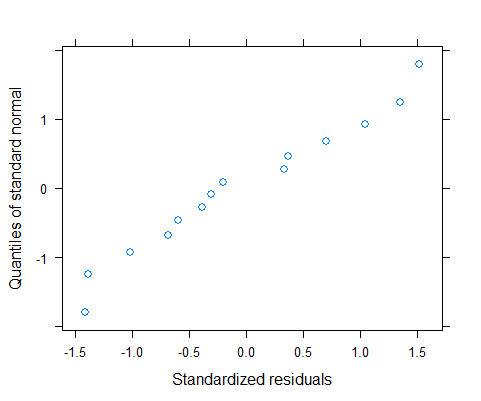


**Diagnostic plots for** Model S-XV.

Standardised residuals by fitted values and normal quantile-quantile plot of standardised residuals.

## Model S-XVI

The model shown in S9 Fig (all strains) fits log_2_ mutation rate against log_2_ effective population size (*N_e_*), treatment (genotype/marker combination) and their interaction and random effects on the intercept of plate nested within block. Variance changed with genotype and [*D*]^-0.18^. Further details are given in ANOVA table and in diagnostic plots.

**ANOVA table and fitted values for** Model S-XVI **(**S9 Fig**).**

See Materials and Methods for more details.

|  | Degrees of freedom | Value | SE | F | P |
| --- | --- | --- | --- | --- | --- |
| Intercept (BY4742) | 1 | -3.8 | 2.6 | 2038 | 0.15 |
| log2(*N_e_*) | 1 | 0.67 | 0.20 | 185 | 6.9×10^-4^ |
| genotype_marker  mutT2_nalidixic_acid30 | 19 | 8.9 | 3.6 | 62 |  |
| nei_rifampicin50 |  | 17 | 5.6 |  |  |
| metI_rifampicin50 |  | 28 | 8.6 |  |  |
| MG1655_rifampicin50 |  | 12 | 5.1 |  |  |
| MG1655_nalidixic_acid30 |  | 12 | 9.5 |  |  |
| PAO1_rifampicin50 |  | 3.0 | 6.0 |  |  |
| S288C_5-fluoroorotic_acid1000 |  | -3.8 | 6.8 |  |  |
| BY4742_hygromycinB300 |  | 15 | 4.2 |  |  |
| Sigma_1278b_hygromycinB300 |  | 7.7 | 4.6 |  |  |
| mutH_nalidixic_acid30 |  | 10 | 3.2 |  |  |
| mutL_nalidixic_acid30 |  | 5.9 | 3.6 |  |  |
| dinB_rifampicin50 |  | 9.8 | 5.1 |  |  |
| mutS_nalidixic_acid30 |  | 7.2 | 5.0 |  |  |
| dam_rifampicin50 |  | 21 | 8.9 |  |  |
| mutM_rifampicin50 |  | 7.4 | 5.3 |  |  |
| mutY_rifampicin50 |  | 5.0 | 5.1 |  |  |
| PCD1_by_hygromycinB300 |  | 4.3 | 3.9 |  |  |
| PCD1_sigma_hygromycinB300 |  | 1.7 | 5.2 |  |  |
| MLH1_sigma_5-fluoroorotic_acid1000 |  | 7.9 | 4.3 |  |  |
| log2(Ne):genotype_marker  log2(Ne):mutT2_nalidixic_acid30 | 19 | -0.75 | 0.27 | 2.7 |  |
| log2(Ne):nei_rifampicin50 |  | -1.4 | 0.43 |  |  |
| log2(Ne):metI_rifampicin50 |  | -2.0 | 0.55 |  |  |
| log2(Ne):MG1655_rifampicin50 |  | -0.98 | 0.33 |  |  |
| log2(Ne):MG1655_nalidixic_acid30 |  | -1.1 | 0.51 |  |  |
| log2(Ne):PAO1_rifampicin50 |  | -0.57 | 0.40 |  |  |
| log2(Ne):S288C_5-fluoroorotic_acid1000 |  | 0.29 | 0.52 |  |  |
| log2(Ne):BY4742_hygromycinB300 |  | -0.77 | 0.30 |  |  |
| log2(Ne):Sigma_1278b_hygromycinB300 |  | -0.58 | 0.31 |  |  |
| log2(Ne):mutH_nalidixic_acid30 |  | -0.70 | 0.24 |  |  |
| log2(Ne):mutL_nalidixic_acid30 |  | -0.39 | 0.27 |  |  |
| log2(Ne):dinB_rifampicin50 |  | -0.81 | 0.38 |  |  |
| log2(Ne):mutS_nalidixic_acid30 |  | -0.51 | 0.39 |  |  |
| log2(Ne):dam_rifampicin50 |  | -1.7 | 0.68 |  |  |
| log2(Ne):mutM_rifampicin50 |  | -0.62 | 0.40 |  |  |
| log2(Ne):mutY_rifampicin50 |  | -0.45 | 0.38 |  |  |
| log2(Ne):PCD1_by_hygromycinB300 |  | 0.020 | 0.28 |  |  |
| log2(Ne):PCD1_sigma_hygromycinB300 |  | 0.19 | 0.35 |  |  |
| log2(Ne):MLH1_sigma_5-fluoroorotic_acid1000 |  | -0.74 | 0.31 |  |  |


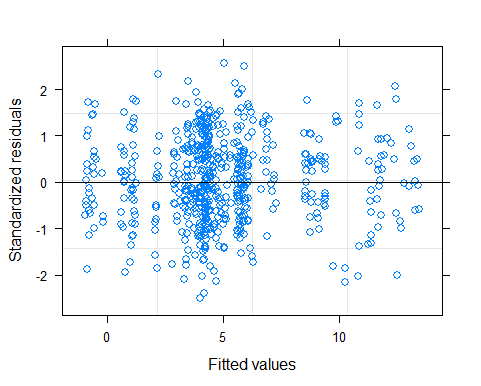

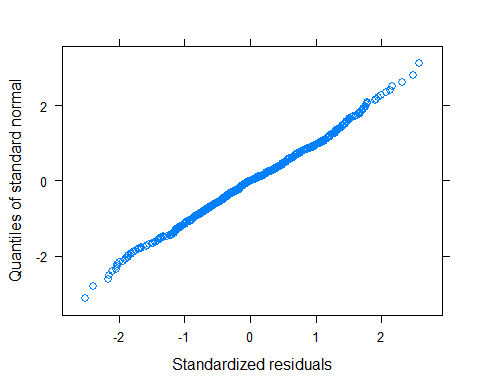


**Diagnostic plots for** Model S-XVI.

Standardised residuals by fitted values and normal quantile-quantile plot of standardised residuals.

## Model S-XVII

The model shown in S10 Fig (all strains) fits log_2_ [number of mutational events] / [culture volume x culture time] against log_2_ *D*, treatment (genotype/marker combination) and their interaction and random effects on the intercept of plate nested within block. Variance changed with [standard deviation of the estimated number of mutational events x selective marker]. Further details are given in ANOVA table and in diagnostic plots.

**ANOVA table and fitted values for** Model S-XVII (S10 Fig).

See Materials and Methods for more details.

|  | Degrees of freedom | Value | SE | *F* | | | *P* | |
| --- | --- | --- | --- | --- | --- | --- | --- | --- |
| Intercept (BY4742) | 1 | -12 | 1.7 | | 732 | 3.0×10^-12^ | |  |
| log2(*Nt*) | 1 | 0.53 | 0.072 | | 42 | 5.3×10^-13^ | |  |
| genotype_marker  dam_rifampicin50 | 19 | 14 | 3.2 | | 54 |  | |  |
| dinB_rifampicin50 |  | 15 | 3.0 | |  |  | |  |
| metI_rifampicin50 |  | 15 | 4.6 | |  |  | |  |
| MG1655_nalidixic_acid30 |  | -3.6 | 4.4 | |  |  | |  |
| MG1655_rifampicin50 |  | 3.7 | 2.7 | |  |  | |  |
| MLH1_sigma_5-fluoroorotic_acid1000 |  | 19 | 7.1 | |  |  | |  |
| mutH_nalidixic_acid30 |  | -1.8 | 2.6 | |  |  | |  |
| mutL_nalidixic_acid30 |  | -5.0 | 2.6 | |  |  | |  |
| mutM_rifampicin50 |  | 17 | 3.5 | |  |  | |  |
| mutS_nalidixic_acid30 |  | -3.4 | 2.8 | |  |  | |  |
| mutT1_nalidixic_acid30 |  | -18 | 2.6 | |  |  | |  |
| mutT2_nalidixic_acid30 |  | -9.6 | 4.0 | |  |  | |  |
| mutY_rifampicin50 |  | 12 | 3.7 | |  |  | |  |
| nei_rifampicin50 |  | 17 | 3.6 | |  |  | |  |
| PAO1_rifampicin50 |  | -15 | 3.7 | |  |  | |  |
| PCD1_by_hygromycinB300 |  | -7.2 | 3.2 | |  |  | |  |
| PCD1_sigma_hygromycinB300 |  | -5.8 | 3.2 | |  |  | |  |
| S288C_5-fluoroorotic_acid1000 |  | 0.020 | 3.6 | |  |  | |  |
| Sigma_1278b_hygromycinB300 |  | 0.89 | 3.5 | |  |  | |  |
| log2(Nt):genotype_marker  log2(Nt):dam_rifampicin50 | 19 | -0.67 | 0.13 | | 17 |  | |  |
| log2(Nt):dinB_rifampicin50 |  | -0.75 | 0.12 | |  |  | |  |
| log2(Nt):metI_rifampicin50 |  | -0.73 | 0.17 | |  |  | |  |
| log2(Nt):MG1655_nalidixic_acid30 |  | -0.26 | 0.15 | |  |  | |  |
| log2(Nt):MG1655_rifampicin50 |  | -0.33 | 0.11 | |  |  | |  |
| log2(Nt):MLH1_sigma_5-fluoroorotic_acid1000 |  | -0.90 | 0.26 | |  |  | |  |
| log2(Nt):mutH_nalidixic_acid30 |  | -0.02 | 0.10 | |  |  | |  |
| log2(Nt):mutL_nalidixic_acid30 |  | 0.087 | 0.11 | |  |  | |  |
| log2(Nt):mutM_rifampicin50 |  | -0.80 | 0.14 | |  |  | |  |
| log2(Nt):mutS_nalidixic_acid30 |  | 0.011 | 0.11 | |  |  | |  |
| log2(Nt):mutT1_nalidixic_acid30 |  | 0.56 | 0.10 | |  |  | |  |
| log2(Nt):mutT2_nalidixic_acid30 |  | 0.19 | 0.16 | |  |  | |  |
| log2(Nt):mutY_rifampicin50 |  | -0.61 | 0.15 | |  |  | |  |
| log2(Nt):nei_rifampicin50 |  | -0.82 | 0.13 | |  |  | |  |
| log2(Nt):PAO1_rifampicin50 |  | 0.26 | 0.14 | |  |  | |  |
| log2(Nt):PCD1_by_hygromycinB300 |  | 0.41 | 0.14 | |  |  | |  |
| log2(Nt):PCD1_sigma_hygromycinB300 |  | 0.36 | 0.15 | |  |  | |  |
| log2(Nt):S288C_5-fluoroorotic_acid1000 |  | -0.19 | 0.15 | |  |  | |  |
| log2(Nt):Sigma_1278b_hygromycinB300 |  | -0.20 | 0.14 | |  |  | |  |


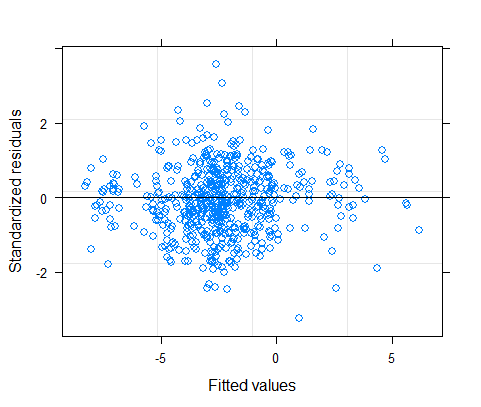

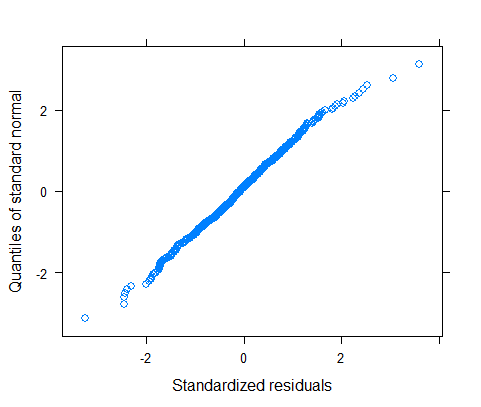


**Diagnostic plots for** Model S-XVII.

Standardised residuals by fitted values and normal quantile-quantile plot of standardised residuals.

## Model S-XVIII

The initial model shown in S11 Fig (Vesicular stomatitis virus***) fits log_2_ mutation rate against mean-centred log_2_ *D* (estimated with plaque forming units, effects of the host cell (host cell/genotype combination) and combined fixed effect of oxygen and temperature. No random effects were included. After removing the interaction no further simplification of the fixed effects was possible without significantly reducing the goodness of fit of the model. Further details are given in ANOVA table and in diagnostic plots.

***Sanjuan R, Nebot MR, Chirico N, Mansky LM, Belshaw R. Viral mutation rates. J Virol. 2010;84:9733-48.

**ANOVA table and fitted values for** Model S-XVIII (S11 Fig).

See Materials and Methods for more details.

|  | Degrees of freedom | Value | SE | | *F* | | *P* |
| --- | --- | --- | --- | --- | --- | --- | --- |
| Intercept (BHK) | 1 | 15 | | 0.26 | | 32471 | 1.9×10^-26^ |
| log_2_(*D*)*_centred_* | 1 | -0.48 | | 0.088 | | 24 | 1.7×10^-5^ |
| host_genotype (C6/36) | 7 | -2.6 | | 0.34 | | 26 | 9.1×10^-8^ |
| (CT26) |  | -0.38 | | 0.31 | |  |  |
| (MEF) |  | -0.18 | | 0.31 | |  |  |
| (MEF_p53) |  | -1.0 | | 0.31 | |  |  |
| (Neuro) |  | -0.32 | | 0.32 | |  |  |
| (S2) |  | -3.5 | | 0.34 | |  |  |
| (sf21) |  | -2.9 | | 0.34 | |  |  |
| oxygen_temperature (normal_28) | 2 | 0.16 | | 0.37 | |  | 0.69 |
| (normal_37) |  | -0.87 | | 0.31 | |  |  |


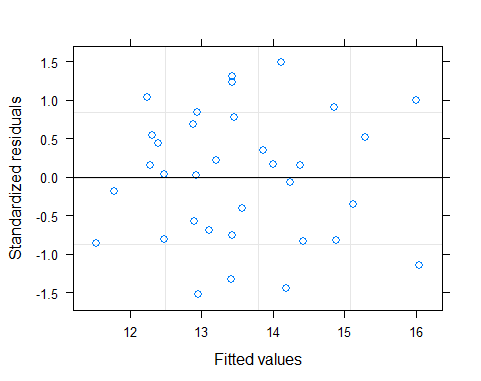

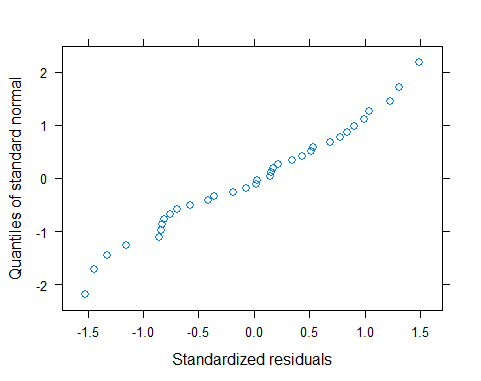


**Diagnostic plots for** Model S-XVIII.

Standardised residuals by fitted values and normal quantile-quantile plot of standardised residuals.
